# What Online User Innovation Communities Can Teach Us about Capturing the Experiences of Patients Living with Chronic Health Conditions. A Scoping Review

**DOI:** 10.1371/journal.pone.0156175

**Published:** 2016-06-07

**Authors:** Julia Amann, Claudia Zanini, Sara Rubinelli

**Affiliations:** 1 Department of Health Sciences and Health Policy, University of Lucerne and Swiss Paraplegic Research, Lucerne/Nottwil, Switzerland; 2 Swiss Paraplegic Research, Nottwil, Switzerland; Utrecht University, NETHERLANDS

## Abstract

**Background:**

In order to adapt to societal changes, healthcare systems need to switch from a disease orientation to a patient-centered approach. Virtual patient networks are a promising tool to favor this switch and much can be learned from the open and user innovation literature where the involvement of online user communities in the innovation process is well-documented.

**Objectives:**

The objectives of this study were 1) to describe the use of online communities as a tool to capture and harness innovative ideas of end users or consumers; and 2) to point to the potential value and challenges of these virtual platforms to function as a tool to inform and promote patient-centered care in the context of chronic health conditions.

**Methods:**

A scoping review was conducted. A total of seven databases were searched for scientific articles published in English between 1995 and 2014. The search strategy was refined through an iterative process.

**Results:**

A total of 144 studies were included in the review. Studies were coded inductively according to their research focus to identify groupings of papers. The first set of studies focused on the interplay of factors related to user roles, motivations, and behaviors that shape the innovation process within online communities. Studies of the second set examined the role of firms in online user innovation initiatives, identifying different organizational strategies and challenges. The third set of studies focused on the idea selection process and measures of success with respect to online user innovation initiatives. Finally, the findings from the review are presented in the light of the particularities and challenges discussed in current healthcare research.

**Conclusion:**

The present paper highlights the potential of virtual patient communities to inform and promote patient-centered care, describes the key challenges involved in this process, and makes recommendations on how to address them.

## Introduction

During the last decades, the world has experienced a demographic and epidemiological transition [1], characterized by an aging population and an increase in chronic health conditions. These changes are associated with increasing costs and poor health outcomes in individuals with chronic health conditions, posing significant challenges for health care systems on a global scale [1,2]. It has been suggested, that in order to fully adapt to societal changes and the resulting consequences for population health, healthcare needs to innovate by switching its focus “from a disease orientation to a patient goal orientation, focused on maximizing the health goals of individual patients” [3].

The involvement of patients in the healthcare innovation process is increasingly recognized as a main asset to favor this switch. Guided by Omachonu’s and Einspruch’s definition of healthcare innovation, we consider the healthcare innovation process to be concerned with the identification and introduction of new concepts and ideas related to services, processes or products that seek to improve treatment, diagnosis, education, outreach, prevention, and research with the ultimate goal of improving health outcomes, quality, safety, efficiency, and cost-effectiveness [4,5]. It is a complex organizational process that is deliberately initiated and entails co-operative and collective activities that are shaped by the individual intentions, preferences, and interests of the different stakeholders involved in and affected by the process and its outcomes [6]. Important stakeholders to be considered in this context are, for example, health professionals, healthcare managers, researchers, but also patients and their families. For the purpose of this paper, we use the term patients for individuals affected by chronic or multiple chronic health conditions who are actual or potential recipients of healthcare.

Indeed, an emerging body of literature documents the value of peer-led self-management support workshops and their potential to improve health literacy and foster patient empowerment [7,8]. Two of the key arguments for using such peer-group approaches are the expected reduction in costs and the potential value of group learning [9]. In addition, patients themselves usually have a great level of experiential credibility when sharing their health related experiences with peers [10]. In this context, the notions of patient-centered care and patient-driven innovation are gaining increasing attention [11–15], even in traditionally highly regulated areas like the pharmaceutical sector [16–18]. As highlighted by Smits and Boon [17], patients can contribute to the healthcare innovation process in a number of ways, for example, by facilitating clinical trials and thereby making the innovation process more efficient or by contributing their own insights and ideas.

In this paper we propose virtual patient networks as a promising tool to favor and promote the active involvement of patients in the innovation process. Health-related online communities have in fact become a main platform for patients to exchange and discuss their health related experiences [19]. However, and despite their increasing popularity, the innovative potential of these virtual patient networks to function as a tool to integrate the patients’ perspective into the healthcare innovation process remains under investigated [20]. Indeed, previous research on health-related online communities has predominantly focused on investigating usage behavior [21–23] and the impact of web-based communities on individuals’ health and social outcomes [24–29]. So rather than reviewing the healthcare literature, we chose to draw on research findings originating from different scientific disciplines to investigate their transferability and applicability to the healthcare context.

By reviewing the open and user innovation literature where the active involvement of online user communities in the innovation process is well-documented, we seek to leverage existing evidence from other fields to inform healthcare research and practice. We believe that this evidence can be a main source of information and innovation to foster the development of virtual patient-innovation communities. The idea underlying open innovation is that firms should leverage internal as well as external sources of innovation and commercialization to capture value and maximize economic profit [30,31]. Research in this field is concerned with firms’ approaches to access external knowledge and innovation and with mechanisms involved in firms’ outward transfer of innovation for commercialization by others [32]. Research in the field of user innovation, on the other hand, is more concerned with the process of value creation by users, investigating tools, processes, and policies that favor such innovation and its diffusion [33,34]. Online communities have become a widely studied phenomenon in this stream of research and also firms are increasingly interested in using them as a tool to integrate users’ ideas in the product and service development process. In this context, Grabher and Ibert [35] distinguish three types of virtual communities: firm-hosted, firm-related, and independent online communities. Firm-hosted communities are those communities initiated, maintained, and governed by commercial producers. The second type, firm-related communities, despite being associated with a brand or product, are initiated and governed by community members in a self-organized process. Contrary to the first two types, independent communities have no link to professional or commercial organizations and are driven solely by the community members and their epistemic goals.

Taking evidence from the study of these online user innovation communities as a starting point, we focuses on the role of virtual patient networks for people with chronic health conditions to foster the integration of the patient’s perspective into the healthcare innovation process. The objectives of this paper are to 1) describe the use of online communities as a tool to capture and harness innovative ideas of end users or consumers in different fields; and 2) point to the potential value and challenges of these virtual platforms to function as a tool to inform and promote patient-centered care in the context of chronic health conditions. The paper proceeds as follows: In the next section, we present our methodological approach, describing our search strategy and analytical procedure. In the results section, we address our first research objective by providing a narrative account of the included studies. The discussion section of the paper then addresses our second research objective by discussing the results of the scoping review in the light of the particularities present in the healthcare context. In the discussion section, we also draw attention to promising directions for future research and acknowledge the limitations of our study. We conclude by highlighting the contribution of our work and its implications for healthcare research and practice.

## Methods

In seeking to link the available evidence from the open and user innovation literature to the healthcare context, we expected a highly heterogeneous and methodologically diverse body of literature [36]. As prior healthcare research has successfully demonstrated the ability of the scoping review methodology to yield a meaningful account of existing research spanning multiple disciplines [37], we chose to follow this approach and carried out a scoping review, allowing us to capture the breadth and depth of available evidence regardless of study design [38,39]. To ensure a rigorous approach, our research was guided by the five-stage framework for conducting scoping reviews developed by Arksey and colleagues [40] and followed PRISMA reporting guidelines [41] (see: S1 PRISMA Checklist). In the following paragraphs we describe our procedures at each of the five stages of the scoping review framework. The detailed review protocol is available (see: S1 Protocol).

In a first step, the research team identified a number of questions based on a preliminary review of the literature, which resulted in two central research questions: What do we know about the use of online communities as a tool to capture user insights in other fields? And to what extent are these findings transferable to the healthcare context? We then proceeded to the identification of relevant studies. The literature search was performed through the databases ISI Web of Science, Communication & Mass Media Complete, PsycINFO, Business Source Premier, MEDLINE, ABI Inform Global, and ABI Trade & Industry. These databases were chosen to cover a broad range of social sciences content. The search strategy was developed based on a preliminary literature review, one lead to the identification of terms relating to open and user innovation in online communities. The full search strategy is described in more detail in the review protocol (see: S1 Protocol). Articles published in English between 1995 and 2014 were taken into consideration. All the references identified, including abstracts, were exported to EndNote.

After de-duplication, the remaining records (n = 11868) were converted to RIS format and imported into EPPI reviewer 4, a web-based software tool for research synthesis. In a next step, the research team proceeded to select relevant studies. Inclusion and exclusion criteria were refined through an iterative process comprised of three levels of relevance screening. The first level of screening comprised a review of title and abstract to eliminate items that did not meet our inclusion criteria. Two coders were involved in the screening process. Inter-coder reliability was assessed for 20% of the records and yielded 90% correspondence. 8754 records were discarded at this first stage. Upon completion of a second round of title and abstract screening, 426 articles were retrieved for full text screening. To be eligible for the review, studies had to be published in English between 1995 and 2014 and describe the user innovation or co-creation process taking place within one or more firm-hosted, firm-related or independent online user innovation communities. For the purpose of this study, we defined online user innovation community as a virtual gathering of end users engaging in product and service development related tasks and activities, such as proposing ideas, evaluating and commenting others’ ideas for product or service improvement, or prototyping. Studies were excluded if they examined online communities that did not involve user innovation activities (e.g. dating communities) or that did not focus on end-user (consumer) involvement but rather on employee or supplier integration. Studies reporting on offline initiatives to integrate consumers in the innovation process, studies investigating innovation intermediaries or crowdfunding practices were also excluded.

Once we had identified and selected relevant studies, data-charting form was developed by the research team to determine which data to extract. As described by Arksey and O’Malley [40], we used a charting approach similar to a narrative review, also referred to as descriptive-analytical method [38], that was guided by our central research questions and the individual sub-questions formulated in the first stage. In addition to extracting descriptive study characteristics, we also recorded information on the original research results and the authors’ conclusions [37]. The complete data charting form is presented in S1 Protocol. Finally, the data were compiled in a single spreadsheet for coding and analysis. We first performed a descriptive summary analysis followed by a qualitative thematic analysis of the data [38]. This helped us to categorize the studies according to their research focus. One author completed the majority of the coding and analysis. From this analysis three categories of studies were coded, according to whether they addressed “user and community factors”, “organizational factors”, or “output and outcome”. One study focused on describing an internet-mediated netnography approach [42]. Although these categories were neither comprehensive nor mutually exclusive, they provided a useful framework to structure the analysis and presentation of results. Table 1 shows the distribution of articles according to the respective categories (see also: S1 Table).

**Table 1:** Distribution of studies according to category.

Category	N (%)
User and Community Factors	82 (56.9%)
Organization Factors	81 (56.3%)
Output and Outcome	50 (34.7%)
Other	1 (0.7%)

## Results

**Fig 1:**
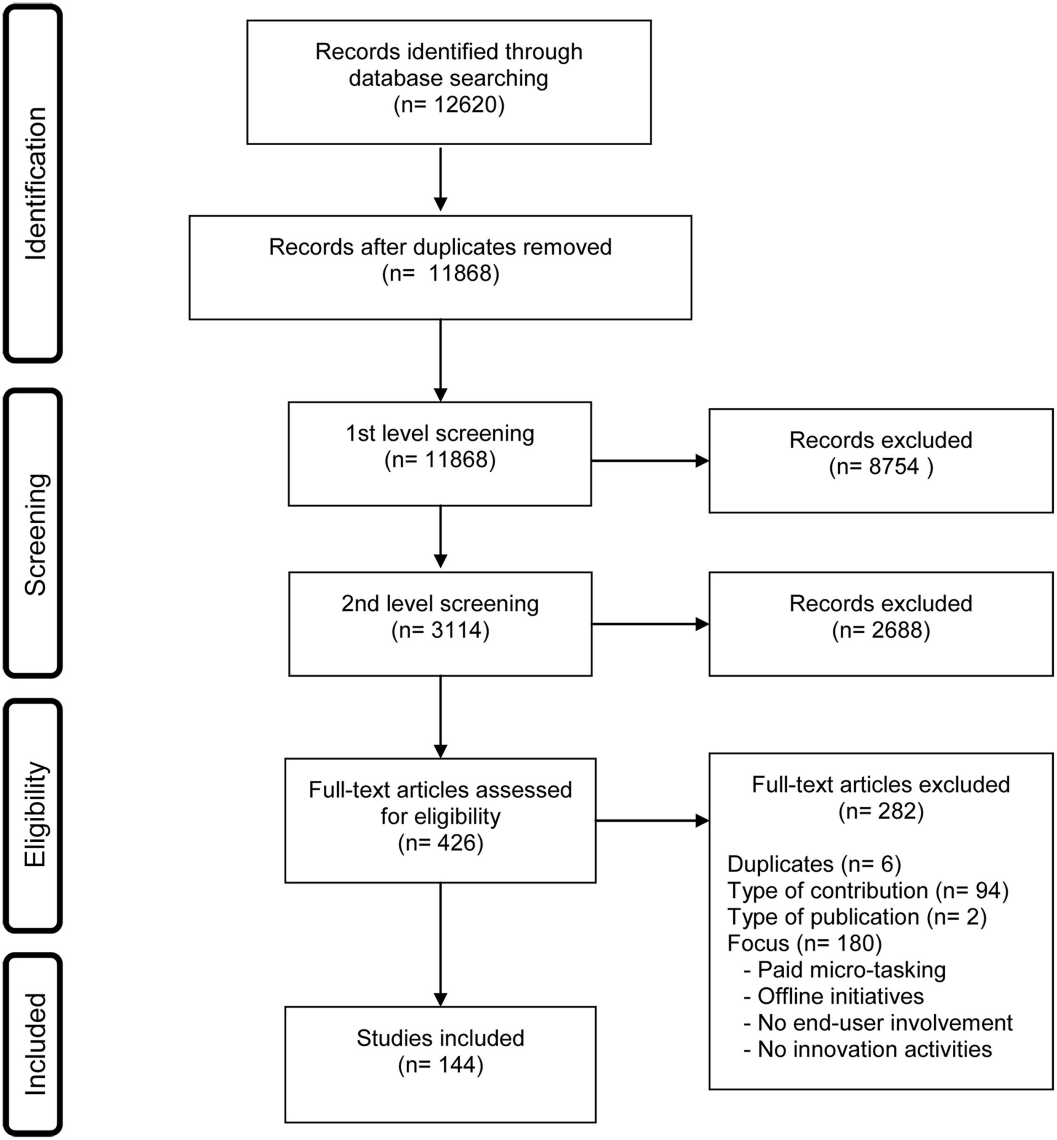
Flowchart.

A total of 144 original studies were included (Fig 1). There has been a notable increase in related publications from the year 2000 until 2014, highlighting the growing interest in online open innovation communities. More than half of the articles included in our study were published between 2012 and 2014 (n = 86). The fact that a noteworthy amount of studies included in this review were published in practitioner journals, such as the MIT Sloan Management Review, further showed that the topic has also been taken up by managers and business executives. Table 2 provides an overview of the most represented journals, highlighting the dominant position of the management literature in the field.

**Table 2:** Most represented source journals.

Name of Journals	Number of articles
	n (%)
MIT Sloan Management Review	8 (5.6)
Organization Science	8 (5.6)
Innovation Management Policy & Practice	5 (3.5)
International Journal of Innovation Management	5 (3.5)
Information Systems Research	4 (2.8)
Journal of Management Information Systems	4 (2.8)
Journal of Product Innovation Management	4 (2.8)
Management Science	4 (2.8)
Internet Research	3 (2.1)
R&D Management	3 (2.1)
Research Policy	3 (2.1)
Research Technology Management	3 (2.1)
Technovation	3 (2.1)
	57 (39.6)

Most of the included studies addressed user innovation initiatives in the private industry [43–48] with a few exceptions from the public sector [49–55]. They were characterized by a great degree of heterogeneity in terms of their research focus, setting, and methodological approaches. Many studies followed a single or multiple case study approach [56–62]. The included studies employed a broad variety of different quantitative as well as qualitative methods such as interviews, surveys, social network analysis, netnography and observation [63–70]. In the following sub sections sub-sections we present the evidence identified in each of the three study categories described earlier, namely “user and community factors”, “organization factors”, and “output and outcome”.

### User and Community Factors

The first category included articles focusing on factors related to the individual users of the platform and the community as a whole. In this context, the studies examined the interplay of different aspects related to individual and community roles, behaviors, and interaction that contribute to the functioning of an open innovation community. In this section, we present findings related to user motivations and factors influencing users’ contribution behavior in virtual innovation communities as well as findings concerning user interaction and the structure of virtual communities.

#### User Motivations

For the purpose of this review, we are guided by the distinction as proposed by Raasch and von Hippel, who refer to output-related and process-related motivations [71]. Output-related motivations refer to benefiting from the innovation itself either through personal use or through potential profit. Process-motivated individuals are motivated by factors, such as the enjoyment and the personal satisfaction they derive from helping other community members [71]. Motivational drivers are considered the strategies and environmental conditions that facilitate and drive individuals’ motivations to engage in knowledge-sharing behavior [72]. Following Battistella and colleagues, we define the environment as the online platform [72], considering a) the platform design characteristics, b) the managerial actions performed by community managers, and c) the interaction between the members inside this platform to be motivational drivers.

The papers included in this review addressed motivational factors in a variety of different settings ranging from open source software development [73–75] to consumer goods such as clothing and accessories [76–78], identifying user motivations to actively contribute [79–82] as well as motivations to collaborate and jointly develop with other community members [57,83,84]. Authors further investigated the link of motivations to different motivational drivers, user types and roles [45,85–89], and different types of co-creation activities and tasks [76,78,90–92]. User motivations and motivational drivers affecting user participation in online open innovation communities were found to be highly context dependent [84,93,94]. Users were usually motivated by a mixture of both output-related and process-related factors which were enforced by a set of motivational drivers [73,76,84,90,95–97]. Process-related individuals primarily sought fun and enjoyment in their participation activities and were eager to learn and develop their skills [71,78,83,95,98]. They were further motivated by gaining recognition from both the hosting firm and other users [46,95,96,99], whereas output-motivated individuals had an actual need for the innovation itself or were seeking some kind of reward (e.g. cash prize) related to their participation [51,71,90]. Studies included in the review also showed that motivational factors were linked to (a) the type of user and their inherent goals, experiences, needs and perceptions [51,79,100–103], (b) the different types of co-creation formats [57,104], and (c) the nature of the co-creation tasks [71,83,105]. Usability aspects, for example, were found to be an important motivational driver for new members joining a community, however, once acquainted with the technical aspects did not any longer affect user motivations to contribute [79]. Authors have also found evidence suggesting that by rewarding individual contributors rather than teams, platforms managed to increase participation, but not collaboration [57]. With respect to the nature of co-creation tasks, authors suggested that various users might find different tasks or problems particularly intriguing which will in turn determine their contribution behavior [71].

The key inference drawn from this section was that user motivations in virtual communities are complex, heterogeneous, and highly context dependent. The reviewed literature did not provide clear directions with respect to the importance or significance of individual motivational factors but rather highlighted a number of aspects that shape user contribution behavior in virtual innovation communities.

#### User Interaction and Community Structure

Studies also examined different user characteristics and roles, investigating ways to identify different user types (such as user innovators and community leaders) based on contribution behavior and roles taken on within the community [48,80,106–109]. The key inference drawn with respect to user interaction and community structure was that despite the size of an online community, usually a small core community of active participants was responsible for the majority of contributions within the platform [110] as there was often a lack of highly involved members who stayed active over longer periods of time [111]. Martins and colleagues suggested that this may be in part due to the fact that members viewed platforms as interactive company web sites where they can get information, rather than as a collective of people with a common interest or passion [111]. So despite constantly observing the platform activity, they tended not to contribute much. Similarly, Füller and colleagues found that over half of the users in their study could be characterized as lurkers, who the authors described as registered members who do not hold social ties to other members, and rarely contribute but rather observe and read interesting discussions. The authors underlined the fact that also numerous unregistered lurkers are to be expected as passive observers of online communities [46]. In this context, the relevance of self-selection for crowdsourcing activities was highlighted as it may lead to better solutions [48,78]. Füller and colleagues argued, that contrary to other scientific research where self-selection is primarily regarded as a bias, the fact that only certain individuals join and participate in open innovation platforms can be seen as an advantage for idea competitions as it helps to overcome local search bias and ensures participants’ intrinsic motivations [78].

Further, studies shed light on the knowledge creation process by identifying different kinds of contributions and contribution activities as well as their specific role in the value creation process [35,84,112–115]. A clear inference was that there were associations between user interaction, user contribution behavior, user performance and the generated output [64,84,113,116–122]. Our findings indicated that particularly collaborative efforts were successful in creating valuable output in terms of idea quality [84,95,114,122]. It has also been suggested that a combination of collaboration and competition elements may be a promising approach [102,123]. In this context, Kosonen and colleagues suggested that collaborative efforts are presumably related to idea quality, whereas competitions are considered to lead to an increase in the quantity of submitted ideas [95]. Blohm and colleagues, for example, showed that user collaboration had a positive influence on idea quality, suggesting that promoting user collaboration could render idea competitions more effective. Similarly, Sigala proposed that it was not the number of submissions but rather the user interactions and discussion that evolved around the submitted ideas that reveal the richest information, as reading about others’ experiences resulted in discussions that helped customers to identify and propose more successful ideas. Other factors enhancing idea generation were the variety of roles users assume, the heterogeneity of the community, and the moderating role of company employees triggering discussions and engaging with the community [47,114,124]. Authors also investigated specific structural characteristics of open innovation communities and communication patterns [110,125–132]. By examining the effect of specific network structures, Singh, for example, showed that small-world properties of an online community led to successful projects in terms of code development as well as user acceptance. The author argued that small-world communities, which are characterized by a higher degree of interconnection and by persons who know each other well through collaboration or common links, and the network size had an impact on project success [126].

Findings from the studies reviewed in this section drew attention to the fact that virtual innovation communities are usually run and dominated by a small, active core community of self-selected individuals. Contrary to clinical research, authors did not view self-selection as a bias in this context but rather an advantage, acknowledging that not all users will make valuable contributions. The findings presented in this section also highlighted the importance of interaction between users to enable knowledge creation, for example in form of product-related discussions. Finally, they also demonstrated how firms can adopt an active role in this process by moderating and triggering discussions.

### Organizational Factors

The second category comprised studies focusing on aspects related to firms’ strategy, such as the design and functionalities of the open innovation platform [57,79,81,90,133], as well as the mode and frequency of interaction between firm and community [134–137]. Studies in this set also addressed organizational challenges and the lessons learned associated with the implementation, maintenance, and exploitation of an online open innovation community [44,138,139].

One of the key findings was that only a small percentage of initiatives aiming at eliciting user contributions managed to do so successfully [101], as many of them failed to generate solutions with competitive advantage potential [104]. Another central claim emerging from the included studies was that open innovation communities are a resource intensive endeavor and not a free or low-cost alternative to internal R&D activities [138]. Companies had to invest a significant amount of resources in time consuming activities related to the initiation and maintenance of an online open innovation community [140]. In addition, the included articles identified different managerial challenges that arise at the various stages of the innovation process [44,136,141,142]. The reluctance to embrace external sources of knowledge due to a fear of leaking proprietary information, loss of managerial power, and ceding control over firm activities, for example, has been shown to limit a company’s ability to fully utilize online communities as a tool for R&D [141].

The studies reviewed investigated a variety of different practices for involving end users in the innovation process including toolkits for user innovation [98,143–145], product platforms [146], open source software communities [137,147,148], and idea and design competitions [96,115,122,123,135]. Studies focused both on autonomous user-led [46,149] as well as firm-sponsored online user communities [47,150]. One study aimed at classifying practices according to their focus, namely technology-oriented and product-oriented practices [151]. Some studies were carried out over several years and went into great detail when describing the design features as well as the underlying processes of the respective platform(s) under investigation [43,152,153]. Two particularly well documented cases were the Dell IdeaStorm [35,45,64,66,106,154,155] and Lego’s user innovation initiatives [44,152,156]. Platform structure in terms of its usability and playfulness was identified as an influential factor when cultivating an active user community [57,79,81,90,133,157]. Further, the availability of tools that facilitate the idea generation process and user interaction was shown to be a promising approach to attract users [47,57,114,157]. In their study Brabham and colleagues even found that members of a user design community reported an “addiction” when referring to their activity of using a design toolkit [76]. Regarding the relationship between company and the user community, the reviewed studies underlined the importance of ensuring a win-win situation for both firm and user community [152]. Factors such as balance between control and autonomy [158–163], company involvement [134–137] and responsiveness [154,160], as well as trust [164] were regarded as crucial for the success of online user innovation initiatives. In their study, Henttonen and colleagues, for example, found that a deeper level of involvement between companies and open source software communities enabled the exchange of more than code, namely ideas, influences, and opinions [136]. Also users’ perception of being treated fairly by the firm, both with respect to the process of idea selection as well as the sharing of profits and recognition, was found to be a critical element [100].

Studies reviewed in this section focused on managerial challenges encountered when collaborating with virtual user communities, acknowledging that the integration of end users in the innovation process is a resource intensive endeavor that requires a clear strategy. Findings presented in this section also pointed to different forms and opportunities to integrate virtual user communities and highlighted the importance of ensuring a win-win situation between the virtual community and firm.

### Output and Outcome

For the purpose of this study, we refer to output as the ideas generated by the user community, whereby we consider outcomes to be direct and indirect market outcomes related to the implementation of these ideas. Studies in this third category investigated processes related to idea selection, implementation, and the resulting market outcomes.

Regarding idea selection, we found that there were various modes and tools for selecting ideas, such as involving users or expert juries in the rating and ranking process [115,156,158,165,166], software tools [167], and computational algorithms [168,169]. Some authors relied on subjective rater assessment of idea quality [170], while others assessed criteria such as novelty, feasibility, relevance, and elaboration [84,171,172]. Others suggested criteria such as idea quantity and idea diversity [102,173–175] or the fact that ideas were implemented [45]. In case of collective code development in open source software communities, the outcome was measured in the code’s performance in addressing a specific problem [73].

Most of the included studies highlighted the success of open innovation communities in terms of their ability to generate valuable ideas and solutions. Authors, for example, compared the ideas generated within an online community to those resulting from traditional forms of market research showing that the use of social media tools for ideation led to more and higher quality ideas at a lower cost per idea in a shorter period of time, as compared to traditional forms of market research [82,141]. Only few authors provided contradictory evidence questioning the role of user communities as successful idea acquisition mechanism [176]. Main points of criticism included that the number of ideas acquired is often low compared to the number of ideas submitted and that great effort and resources are needed to sustain an open innovation community [177]. Studies also reported on outcomes of online user innovation initiatives such as firms’ innovation effectiveness [178], diversification or extension of product portfolio [165,179,180], reduced time to product release [125], improved customer-relations and increased customer-sensing and responding capability [150,181], and outcomes related to brand strength [59,182]. Findings further indicate that whether or not an open innovation community can be successful in generating innovative and profitable ideas that ultimately lead to direct and indirect market outcomes, depended on numerous factors such as the respective industry, company characteristics, managerial attitudes, and corporate strategy [101,163]. Most of all, it depended on the firm’s ability to build and sustain an active community, and its ability to effectively collaborate with users across organizational boundaries [101].

Findings from the studies reviewed in this last section showed that online communities can in fact be an appropriate tool to help firms generate, identify, and collect end user ideas, when used appropriately. Authors also illustrated different approaches for identifying and selecting promising ideas generated by online communities. The reviewed studies further showed that the involvement of virtual communities can in fact positively affect firm performance with respect to the product and service innovation process but also with respect to customer relations.

## Discussion

By reviewing the open and user innovation literature, this review described the use of online communities as a tool to capture and harness innovative ideas developed and shared by end users in different fields. We now seek to link these findings back to the healthcare context, presenting them in the light of the particularities and challenges discussed in current healthcare research. We thereby aim to point to the potential value and challenges of these virtual platforms to function as a tool to inform and promote patient-centered care in the context of chronic health conditions. In order structure this section in a meaningful and pragmatic way, we were guided by the innovation management literature, describing models of product and service innovation and knowledge integration as stage processes [183,184], in particular the ones proposed by Wallin and von Krogh [185] and Alam and Perry [186]. Based on these frameworks and the findings from the review we identified four stages that are critical when involving online user communities in the healthcare innovation process:

1. Setting the stage for online user innovation initiatives
2. Attracting and maintaining an active user community
3. Selecting and absorbing ideas
4. Process and outcome evaluation

Our findings suggest that the operation of open innovation communities is highly context dependent [54,94,102,115,142,187]. So despite the success stories of user innovation initiatives in transforming the innovation processes in various industries [93], important questions arise for the applicability to the healthcare context [188]. This is why, when interpreting our findings, the particularities and resulting challenges of the healthcare setting have to be examined specifically. In the following sections we highlight the key issues for introducing virtual patient networks to the healthcare setting. We do so by pinpointing the critical challenges encountered at each of the stages we identified previously, make recommendations on how to address them, and propose ways to think about user innovation in healthcare contexts, particularly for those living with chronic health conditions.

### Setting the Stage for online User Innovation Initiatives

Before investing in an online platform, it is, first of all, important to consider the significant amount of resources required to initiate, maintain and harness an open innovation community [106,138]. Secondly, our findings suggest that to define and agree on goals and expectations of an online user innovation initiative, it is crucial to get buy-in from all the key stakeholders involved in or affected by the process [43,51,150,189]. Transferring these points to the healthcare context we foresee challenges arising particularly from a) the heterogeneity of the healthcare setting in terms of the different actors with their diverse rationales [190–195], b) the dominance of the top-down, paternalistic approach to care [196–198], and c) the fact that evidence on successful online user innovation initiatives in healthcare is still scarce [199]. Matters are further complicated by strict norms and legal regulations in healthcare, for example with respect to data protection, that vary between and sometimes even within countries [188].

The first point is best illustrated by a concrete example. Taking a hospital for instance, we expect different actor groups such as nurses, surgeons, hospital managers and patients to have different priorities, expectations, and perceptions related to the healthcare process [190,193,196,200]. In their study, Ferrand and colleagues, for example, showed that even though physicians and nurses agreed on standards for collaborative decision-making, there was a strong discrepancy in their perception of the actual process [200]. But particularly patients’ priorities and expectations may not always align with those of healthcare providers [194,195,201]. In this context, Rubinelli and colleagues point to the challenge of patients’ multiple goals, among which health may be one, but not necessarily the most important one [194]. This is illustrated by the example of Lisa Crites, a breast cancer patient who developed a shower shirt to protect herself, but also other mastectomy patients, from post-surgical infection, recognizing that women will want to shower despite the risk of infection [11]. This example shows that the patient may perceive showering as equally important as preventing post-surgical infection. Particularly individuals living with chronic health conditions tend to develop strong views and perceptions with regard to their health condition and treatment [202–204]. Health professionals, however, often view their patients through the lens of medical expertise adopting a “disease focus” rather than recognizing these individual differences.

The second challenge, the dominance of the top-down, paternalistic approach to care, is very closely related to the first one. We assume that there is an imbalance in power due to the dominance of the top-down approach in healthcare [196–198], which impedes successful collaboration and co-creation between patients and physicians. There is, for example, significant evidence suggesting that health professionals perceive the increasing trend of online health information as a threat to their control and medical authority, some even adopt strategies to discourage or undermine patients’ online information seeking efforts [205–207]. This leads us to assume, that patients who start taking matters into their own hands by developing solutions to their health problems may be perceived as even more threatening.

Having addressed the challenges related to the different actors in the healthcare setting, we now turn to the third challenge: the lack of best practice approaches. Despite successful examples from other fields [102,152], little evidence documents strategies for and outcomes expected from successfully applying these principles in healthcare [199]. When viewing user innovation in the light of evidence-based medicine [208], it seems intuitive that healthcare providers are not willing to invest time and money in a tool without having an idea about the achievable outcome. This is why we suggest that successful initiatives from other fields can and should be used to inform goal setting and strategy development in healthcare. Much can be learned, for example, from the open and user innovation practices of Lego, a maker of children’s creative construction toys with a large adult-fan community [44,152,156]. Lego used to be an extremely private (‘closed’) company with high level of product and intellectual property control. But when users started hacking and modifying the software of a new product line, the Lego Mindstorms, Lego had to decide whether to pursue legal action or collaborate with the adult fan community who had started sharing their adapted product versions through independent websites. Lego decided to open up its formerly closed innovation process and invited users to jointly innovate [152].

Drawing parallels to the healthcare setting we observe similar patterns. Departing from the paternalistic ‘closed’ approach of healthcare, patients have started to develop their own solutions to health problems they are facing. Since pursuing legal action against these patients is not a viable option, healthcare providers will have two options: a) Shut patients out and exclude them from the healthcare process taking the risk that they will create their own solutions anyways, or b) Include patients in the healthcare process and make them partners. The rising trend of shared decision-making and patient empowerment [209] suggests that providers will most likely opt for the latter. Future research is needed to make more specific recommendations on how the different perspectives, priorities, and expectations can be aligned taking also resource-constraints into consideration. We propose the process known as ‘stakeholder dialogue’ as a suitable method for achieving this aim. “Stakeholder dialogues allow research evidence to be brought together with the views, experiences and tacit knowledge of those who will be involved in, or affected by, future decisions about a high-priority issue” [210], with the goal of generating action [211].

### Attracting and maintaining an active User Community

So far we have discussed issues related to setting the stage for online user innovation. We now shed light on the challenges related to attracting and maintaining an active user community. From the literature reviewed in this study we identified five key elements that are crucial to the initiation and maintenance of an open innovation platform.

1. Users willingness to share their innovative ideas [46,47,70,136]
2. Attractiveness and usability of the platform [51,57,79,81,90,133]
3. Motivations [71,73,76,84,90,95,96]
4. Interaction and Involvement [45,47,70,137,172]
5. Partnership between user community and firm [81,91,93,95,99,152,156,159,164,165]

When looking at the literature on health-related online communities, we find that the evidence on attracting and maintaining active user communities is scarce. However, authors have shown that patients are not only willing to share their personal health information for research [212,213], but that they are also willing to share their knowledge and solutions with other patients [19,214,215]. Sharing details about their experiences with their health condition, treatment, and the health system help these individuals cope with the struggles they are facing in their everyday life [216–218]. This first element is also the most crucial because if patients are not willing to share their ideas and solutions for reasons such as privacy concerns or fear of stigma, there would be no one to collaborate with.

Related to the second element, we find that in terms of the platform technology, reliable technology seems to be preferable over state-of-the-art technology [20]. This aspect did not emerge from our review, suggesting that it might be a particularity of the healthcare context. A possible explanation for this is that patients may experience a stronger need for a stable and reliable environment which safeguards their personal health information ensuring privacy and security [219].

With respect to the third element, there is very limited understanding of patients’ motivations to share their experiences online. This is partially due to the fact that online communities are frequently designed as an intervention tool tested on randomly assigning participants that were recruited for the study with the purpose of assessing health outcomes [24]. This, however, makes it difficult to assess whether these patients would have actually joined or contributed to an online community without being told to do so. By surveying community members of the PatientsLikeMe platform, Wicks and colleagues avoided this bias and found that users perceived the greatest benefit of the online platform in learning about symptoms and side effects of their treatments. Also, connecting to other patients was considered as beneficial. Some patients perceived the site as helpful when making decisions regarding their medication [213]. This suggests that patients are both output and process-motivated. In other words, patients will, on the one hand, have an inherent interest in solutions to their problems. On the other hand, we also expect partially process-motivated individuals for whom participation constitutes part of their coping strategy [218]. One of the most striking examples in this context is Tal Golesworthy, a process engineer who was diagnosed with an inherited disorder of aorta, a condition which results in a decreasing functionality of the aorta. Instead of accepting the available treatment which entailed lifelong drug therapy, he developed a more suitable solution which eliminated the need for drug therapy. In 2004, Tal was the first patient to have the External Aortic Root Support implanted. The operation is now performed in the UK and Belgium [11]. Of course not all patients come up with innovative and game-changing solutions that they share with others. As observed in other fields, active contributors constitute a clear minority in health communities [218]. In this context, three questions arise: Who are the active patients that develop and share their solutions? What motivates them? And how can we identify them? Even though we cannot answer these questions within the scope of this review, they provide interesting avenues for future research. Scholars should further investigate the characteristics of patient-innovators and their underlying motivations in the context of different health conditions [220].

When addressing the fourth aspect in the healthcare setting, we encounter a key challenge that did not emerge from the studies included in the review: the potential danger resulting from incorrect, incomplete, or simply misinterpreted information and ideas. To illustrate this with an example, imagine a company collaborates with a user community to develop a new MP3 player. Of course the company aims for quality solutions and ideas that will lead to a profitable product, but not all ideas will be good. Even though these bad ideas may prolong the co-creation and idea selection process, there is no harm in bad ideas (as long as they are not implemented). On the contrary, if a person with Type 1 diabetes does not recognize bad ideas as such and implements them, this may pose a serious threat to his or her health. Some patients, particularly those suffering from rare, chronic, or life-threating conditions with no available standard treatments may be desperate for solutions and may be willing to try just about anything [221–223]. This threat becomes more concrete in the light of patients’ suboptimal search strategies and evaluation skills when it comes to online health information [224,225], and in turn raises questions of liability and patient safety [207,226]. Even though healthcare organizations should promote and encourage interaction between users, they should also have clear policies and actively moderate discussions not only to engage individuals, but also to reduce the dissemination of potentially harmful ideas [227,228].

We now turn to the last aspect, the practice of treating users as partners in the innovation process. In the healthcare setting, we propose that despite keeping a certain level of control over community activities, it is important to treat community members as a partners. In other words, they should be recognized as knowledge resources rather than data sources, or passive receivers of care. As pointed out in the introduction of this paper, the lived-experience of people with a chronic condition constitutes an invaluable resource for research and practice. Despite the obvious advantages an online patient community presents for research, such as facilitated data collection and patient recruitment [229], their ability to contribute by sharing their ideas on innovative self-management practices should not be underestimated [19,215].

### Selecting and absorbing Ideas

In the previous section we discussed issues related to the idea generation process. But once ideas are being generated, how can we identify the most viable ones? Findings of our review highlighted various modes and tools for selecting user-generated ideas; however there is no consensus with respect to their effectiveness and efficiency in identifying the most promising ideas [115,156,158,165,168]. Few studies discussed the process of absorbing an internalizing knowledge from the user community [44,150]. This may be due to the switch back to the producer side for commercial exploitation which occurs once the collective exploration with help of the user community is concluded [230].

When discussing the process of idea selection in the healthcare context, issues related to idea quality, that were discussed previously, lead us to the conclusion that in addition to the aspects highlighted by the open and user innovation literature, such as novelty and high customer benefit [170], components of information quality [231–233] may help to develop adequate idea selection criteria. Future research should engage in developing indicators for what constitutes a “good idea” in the healthcare context and investigate methods on how to efficiently identify them. It will also be crucial to develop processes for absorbing patients’ ideas and incorporating them into the care setting. In this context, we refer back to the process known as ‘stakeholder dialogue’ [210,211] as a promising approach to identify and integrate patients’ innovative self-management practices.

### Process and Outcome Evaluation

Most of the included studies highlighted the success of open innovation communities in terms of their ability to generate valuable ideas and solutions. Whether an online user innovation initiative was considered as successful or not was usually measured by the quantity, diversity, and quality of ideas generated [102,129,173]. Central questions for healthcare will be: What are the benefits for the different stakeholders involved? Does participating in an online innovation community help patients better cope with their condition or better self-manage? Do solutions generated through online patient communities improve the quality of care or help to decrease healthcare costs? Future research should address these questions and investigate appropriate indicators for measuring the success of online user innovation initiatives in the healthcare context not only in terms of their ability to generate ideas but also in terms of their impact.

## Limitations

The present paper contributes to the growing body of literature on virtual patient communities, highlighting some of the key aspects relevant to the integration of these communities in the healthcare innovation process. However, some of the limitations should be acknowledged when interpreting our findings. Firstly, due to the heterogeneity of the field in terms of terminology our search strategy, particularly our selection of key words, may have failed to identify some relevant studies. We tried to minimize this risk by conducting a preliminary review of the literature to inform our key word selection and choice of databases. Secondly, the diversity of the included studies with respect to their theoretical framework, study design, and research focus proved to be challenging when seeking to structure the evidence in a meaningful way. We chose to present our findings by grouping studies according to their research focus; however, evidence could have, for example, also been structured according to the type of online community investigated (firm-hosted vs. firm-related vs. independent). Thirdly, we did not account for the quality of the included publications, nor did we account for reporting or publication bias.

## Conclusion

Despite evidence suggesting that there is great potential in harnessing the knowledge and lived-experience of patients and their carers [11,19,198,214], there has been a focus on exploring patient needs rather than on their ability to come up with viable ideas and solutions to their health problems [234]. This paper offers what, to our knowledge, is one of the first attempts to address the innovative potential of online communities by adopting the open and user innovation perspective. It provides a narrative account of the existing literature, describing the use of online communities as a tool to capture and harness innovative ideas of end users or consumers in various fields and points to the potential of these virtual platforms to function as a tool to inform patient-centered care in the context of chronic health conditions. Based on the findings of the scoping review and guided by models used in the innovation management literature, we identified four critical stages in opening up the healthcare innovation process to the input of virtual patient communities. Taking the particularities of the healthcare context into account, we then described the key challenges associated with these stages and made recommendations on how to address them.

In the light of the shift toward patient-driven care, efficient strategies for integrating the ideas and experiences of patients into healthcare research and practice will become crucial. Our findings suggest that much can be learned from online user innovation practices employed in other fields and industries. Future research should further explore these areas and identify strategies to harness the innovative potential of online communities in the healthcare context. It will further be important to identify indicators of success and best practice approaches which will ultimately support healthcare institutions in adopting online communities as a means to integrate patients in the healthcare innovation process.

## Supporting Information

S1 PRISMA Checklist PRISMA 2009 Checklist. (PDF)

S1 Protocol Review Protocol. (PDF)

S1 Table Included studies according to category. (PDF)

## References

[1] Divajeva D, Marsh T, Logstrup S, Kestens M, Vemer P, Kriaucioniene V et al (2014) Economics of chronic diseases protocol: cost-effectiveness modelling and the future burden of non-communicable disease in Europe. BMC public health 14: 456 10.1186/1471-2458-14-456 24886110 PMC4047783

[2] Parekh AK, Kronick R, Tavenner M (2014) Optimizing health for persons with multiple chronic conditions. JAMA 312: 1199–1200. 10.1001/jama.2014.10181 25133982

[3] Tinetti ME, Fried TR, Boyd CM (2012) Designing health care for the most common chronic condition—multimorbidity. JAMA 307: 2493–2494. 10.1001/jama.2012.5265 22797447 PMC4083627

[4] Omachonu VK, Einspruch NG (2010) Innovation in healthcare delivery systems: a conceptual framework. The Innovation Journal: The Public Sector Innovation Journal 15: 1–20.

[5] Greenhalgh T, Robert G, Macfarlane F, Bate P, Kyriakidou O (2004) Diffusion of innovations in service organizations: systematic review and recommendations. Milbank Quarterly 82: 581–629. 15595944 10.1111/j.0887-378X.2004.00325.x PMC2690184

[6] May C, Finch T, Mair F, Ballini L, Dowrick C, Eccles M, et al (2007) Understanding the implementation of complex interventions in health care: the normalization process model. BMC health services research 7: 148 17880693 10.1186/1472-6963-7-148 PMC2089069

[7] Haslbeck J, Zanoni S, Hartung U, Klein M, Gabriel E, Eicher M, et al (2015) Introducing the chronic disease self-management program in Switzerland and other German-speaking countries: findings of a cross-border adaptation using a multiple-methods approach. BMC health services research 15: 1–19. 26711458 10.1186/s12913-015-1251-z PMC4692063

[8] Kennedy A, Reeves D, Bower P, Lee V, Middleton E, Richardson G, et al (2007) The effectiveness and cost effectiveness of a national lay-led self care support programme for patients with long-term conditions: a pragmatic randomised controlled trial. Journal of Epidemiology and Community Health 61: 254–261. 17325405 10.1136/jech.2006.053538 PMC2652924

[9] Newman S, Steed L, Mulligan K (2004) Self-management interventions for chronic illness. The Lancet 364: 1523–1537. 10.1016/S0140-6736(04)17277-2 15500899

[10] Amann J, Rubinelli S, Kreps GL (2015) Revisiting the concept of health literacy. The patient as information seeker and provider. European Health Psychologist 17: 286–290.

[11] Habicht H, Oliveira P, Shcherbatiuk V (2013) User innovators: when patients set out to help themselves and end up helping many. Die Unternehmung 66: 277–294.

[12] Oliveira P, Zejnilovic L, Canhão H, von Hippel E (2014) Patient innovation under rare diseases and chronic needs. Orphanet Journal of Rare Diseases 9: O33–O33. 10.1186/s13023-015-0257-2 PMC4404234 25887544

[13] Kanstrup AM, Bertelsen P, Nøhr C (2015) Patient innovation: an analysis of patients’ designs of digital technology support for everyday living with diabetes. Health Information Management Journal 44: 12–20. 10.1177/183335831504400102 27092465

[14] McNichol E (2012) Patient-led innovation in healthcare: The value of the ‘user’perspective. International Journal of Healthcare Management 5: 216–222.

[15] Spyropoulou GA, Fatah F (2009) Decorative tattooing for scar camouflage: patient innovation. Journal of Plastic, Reconstructive & Aesthetic Surgery 62: e353–e355. 10.1016/j.bjps.2008.01.043 18640088

[16] Boon W, Broekgaarden R (2010) The role of patient advocacy organisations in neuromuscular disease R&D—The case of the Dutch neuromuscular disease association VSN. Neuromuscular disorders 20: 148–151. 20106662 10.1016/j.nmd.2009.10.012

[17] Smits REHM, Boon WPC (2008) The role of users in innovation in the pharmaceutical industry. Drug discovery today 13: 353–359. 10.1016/j.drudis.2007.12.006 18405849

[18] Herxheimer A (2003) Relationships between the pharmaceutical industry and patients' organisations. BMJ: British Medical Journal 326: 1208 12775627 10.1136/bmj.326.7400.1208 PMC1126060

[19] Maloney-Krichmar D, Preece J (2005) A multilevel analysis of sociability, usability, and community dynamics in an online health community. ACM Transactions on Computer-Human Interaction (TOCHI) 12: 201–232.

[20] Bullinger AC, Rass M, Adamczyk S, Moeslein KM, Sohn S (2012) Open innovation in health care: Analysis of an open health platform. Health policy 105: 165–175. 10.1016/j.healthpol.2012.02.009 22440194

[21] Misra R, Mukherjee A, Peterson R (2008) Value creation in virtual communities: the case of a healthcare web site. International Journal of Pharmaceutical and Healthcare Marketing 2: 321–337.

[22] Schulz PJ, Rubinelli S, Zufferey MC, Hartung U (2010) Coping with chronic lower back pain: designing and testing the online tool ONESELF. Journal of Computer‐Mediated Communication 15: 625–645.

[23] Lasker JN, Sogolow ED, Sharim RR (2005) The role of an online community for people with a rare disease: content analysis of messages posted on a primary biliary cirrhosis mailinglist. Journal of Medical Internet Research 7: e10–e10. 15829472 10.2196/jmir.7.1.e10 PMC1550634

[24] Eysenbach G, Powell J, Englesakis M, Rizo C, Stern A (2004) Health related virtual communities and electronic support groups: systematic review of the effects of online peer to peer interactions. Bmj 328: 1166 15142921 10.1136/bmj.328.7449.1166 PMC411092

[25] Høybye M, Dalton SO, Deltour I, Bidstrup P, Frederiksen K, Johansen C (2010) Effect of Internet peer-support groups on psychosocial adjustment to cancer: a randomised study. British journal of cancer 102: 1348–1354. 10.1038/sj.bjc.6605646 20424614 PMC2865756

[26] Houston TK, Cooper LA, Ford DE (2002) Internet support groups for depression: a 1-year prospective cohort study. American Journal of Psychiatry 159: 2062–2068. 12450957 10.1176/appi.ajp.159.12.2062

[27] An LC, Schillo BA, Saul JE, Wendling AH, Klatt CM, Berg CJ, et al (2008) Utilization of smoking cessation informational, interactive, and online community resources as predictors of abstinence: cohort study. Journal of medical Internet research 10: e55 10.2196/jmir.1018 19103587 PMC2630836

[28] Richardson CR, Buis LR, Janney AW, Goodrich DE, Sen A, Hess ML, et al (2010) An online community improves adherence in an internet-mediated walking program. Part 1: results of a randomized controlled trial. Journal of Medical Internet Research 12: e71 10.2196/jmir.1338 21169160 PMC3056526

[29] Johnston AC, Worrell JL, Di Gangi PM, Wasko M (2013) Online health communities. Information Technology & People 26: 213–235.

[30] Chesbrough H (2006) Open innovation: a new paradigm for understanding industrial innovation. Open innovation: Researching a new paradigm: 1–12.

[31] Chesbrough H (2006) Open innovation: The new imperative for creating and profiting from technology: Harvard Business Press.

[32] Lichtenthaler U (2009) Outbound open innovation and its effect on firm performance: examining environmental influences. R&d Management 39: 317–330.

[33] West J, Bogers M (2010) Contrasting innovation creation and commercialization within open, user and cumulative innovation. Academy of Management, Montreal Canada.

[34] Von Hippel EA (2005) Democratizing innovation.

[35] Grabher G, Ibert O (2014) Distance as asset? Knowledge collaboration in hybrid virtual communities. Journal of Economic Geography 14: 97–123.

[36] Dryden R, Williams B, McCowan C, Themessl-Huber M (2012) What do we know about who does and does not attend general health checks? Findings from a narrative scoping review. BMC public health 12: 1. 22938046 10.1186/1471-2458-12-723 PMC3491052

[37] Weeks LC, Strudsholm T (2008) A scoping review of research on complementary and alternative medicine (CAM) and the mass media: looking back, moving forward. BMC complementary and alternative medicine 8: 43 10.1186/1472-6882-8-43 18638413 PMC2494539

[38] Levac D, Colquhoun H, O’Brien KK (2010) Scoping studies: advancing the methodology. Implement Sci 5: 1–9. 20854677 10.1186/1748-5908-5-69 PMC2954944

[39] Davis K, Drey N, Gould D (2009) What are scoping studies? A review of the nursing literature. International journal of nursing studies 46: 1386–1400. 10.1016/j.ijnurstu.2009.02.010 19328488

[40] Arksey H, O'Malley L (2005) Scoping studies: towards a methodological framework. International journal of social research methodology 8: 19–32.

[41] Moher D, Liberati A, Tetzlaff J, Altman DG (2009) Preferred reporting items for systematic reviews and meta-analyses: the PRISMA statement. Annals of internal medicine 151: 264–269. 19622511 10.7326/0003-4819-151-4-200908180-00135

[42] Cromie J, Ewing M (2008) Squatting at the digital campfire—Researching the open source software community. International Journal of Market Research 50: 631–653.

[43] Martini A, Massa S, Testa S (2014) Customer co-creation projects and social media: The case of Barilla of Italy. Business horizons 57: 425–434.

[44] Antorini YM, Muñiz AM Jr. (2013) The Benefits and Challenges of Collaborating with User Communities. Research Technology Management 56: 21–28.

[45] Bayus BL (2013) Crowdsourcing New Product Ideas over Time: An Analysis of the Dell IdeaStorm Community. Management science 59: 226–244.

[46] Füller J, Jawecki G, Mühlbacher H (2007) Innovation creation by online basketball communities. Journal of Business Research 60: 60–71.

[47] Alton YKC, Banerjee S (2013) Customer knowledge management via social media: the case of Starbucks. Journal of Knowledge Management 17: 237–249.

[48] Marchi G, Giachetti C, de Gennaro P (2011) Extending lead-user theory to online brand communities: The case of the community Ducati. Technovation 31: 350–361.

[49] Spiliotopoulou L, Charalabidis Y, Loukis E. N, Diamantopoulou V (2014) A framework for advanced social media exploitation in government for crowdsourcing. Transforming Government: People, Process and Policy 8: 545.

[50] Lee SM, Hwang T, Choi D (2012) Open innovation in the public sector of leading countries. Management Decision 50: 147–162.

[51] Hennala L, Parjanen S, Uotila T (2011) Challenges of multi-actor involvement in the public sector front-end innovation processes. European Journal of Innovation Management 14: 364–387.

[52] Lüders M, Folstad A, Waldal E (2014) Expectations and Experiences With MyLabourParty: From Right to Know to Right to Participate? Journal of Computer-Mediated Communication 19: 446–462.

[53] Evans-Cowley J, Hollander J (2010) The New Generation of Public Participation: Internet-based Participation Tools. Planning Practice & Research 25: 397–408.

[54] Seltzer E, Mahmoudi D (2013) Citizen Participation, Open Innovation, and Crowdsourcing: Challenges and Opportunities for Planning. Journal of Planning Literature 28: 3–18.

[55] Khan Z, Ludlow D, Loibl W, Soomro K (2014) ICT enabled participatory urban planning and policy development. Transforming Government: People, Process and Policy 8: 205.

[56] Latzko-Toth G (2014) Users as Co-Designers of Software-Based Media: The Co-Construction of Internet Relay Chat. Canadian Journal of Communication 39: 577–595.

[57] Antikainen M, Mäkipää M, Ahonen M (2010) Motivating and supporting collaboration in open innovation. European Journal of Innovation Management 13: 100–119.

[58] Spaeth S, Stuermer M, Georg Von K (2010) Enabling knowledge creation through outsiders: towards a push model of open innovation. International Journal of Technology Management 52: 411–431.

[59] Sasinovskaya O, Anderson H (2011) From brand awareness to online co-design: How a small bathroom provider turned interactive on the Web. Journal of Brand Management 19: 33–44.

[60] Nätti S, Hurmelinna-Laukkanen P, Johnston WJ (2014) Absorptive capacity and network orchestration in innovation communities—promoting service innovation. The Journal of Business & Industrial Marketing 29: 173–184.

[61] Raasch C, Herstatt C, Balka K (2009) On the open design of tangible goods. R & D Management 39: 382–393.

[62] Scupola A, Hanne Westh N (2010) Service innovation in academic libraries: is there a place for the customers? Library Management 31: 304–318.

[63] Toral SL, Martinez-Torres MR, Barrero F (2009) Modelling Mailing List Behaviour in Open Source Projects: the Case of ARM Embedded Linux. Journal of Universal Computer Science 15: 648–664.

[64] di Gangi PM, Wasko M (2009) Steal my idea! Organizational adoption of user innovations from a user innovation community: A case study of Dell Ideastorm. Decision Support Systems 48: 303–312.

[65] Toral SL, Martinez-Torres MR, Barrero FJ (2009) Virtual communities as a resource for the development of OSS projects: the case of Linux ports to embedded processors. Behaviour & Information Technology 28: 405–419.

[66] Martinez-Torres MR (2014) Analysis of open innovation communities from the perspective of social network analysis. Technology Analysis & Strategic Management 26: 435–451.

[67] de Toni AF, Biotto G, Battistella C (2012) Organizational design drivers to enable emergent creativity in web-based communities. Learning Organization 19: 337–351.

[68] Hann I-H, Roberts JA, Slaughter SA (2013) All Are Not Equal: An Examination of the Economic Returns to Different Forms of Participation in Open Source Software Communities. Information Systems Research 24: 520–538.

[69] Füller J, Matzler K, Hutter K, Hautz J (2012) Consumers' Creative Talent: Which Characteristics Qualify Consumers for Open Innovation Projects? An Exploration of Asymmetrical Effects. Creativity & Innovation Management 21: 247–262.

[70] Russo-Spena T, Mele C (2012) "Five Co-s" in innovating: a practice-based view. Journal of Service Management 23: 527–553.

[71] Raasch C, von Hippel E (2013) Innovation Process Benefits: The Journey as Reward. MIT Sloan Management Review 55: 33–39.

[72] Battistella C, Nonino F (2012) What drives collective innovation? Exploring the system of drivers for motivations in open innovation, Web-based platforms. Information Research-an International Electronic Journal 17 paper 513. Available: http://InformationR.net/ir/17-1/paper513.html].

[73] Boudreau KJ, Lacetera N, Lakhani KR (2011) Incentives and Problem Uncertainty in Innovation Contests: An Empirical Analysis. Management science 57: 843–863.

[74] Shen C, Monge P (2011) Who connects with whom? A social network analysis of an online open source software community. pp. 39–39.

[75] Xu B, Jones DR, Shao B (2009) Volunteers’ involvement in online community based software development. Information & Management 46: 151–158.

[76] Brabham DC (2010) Moving the crowd at Threadless. Information, Communication & Society 13: 1122–1145.

[77] Hildebrand C, Häubl G, Herrmann A, Landwehr JR (2013) When Social Media Can Be Bad for You: Community Feedback Stifles Consumer Creativity and Reduces Satisfaction with Self-Designed Products. Information Systems Research 24: 14–V.

[78] Füller J, Hutter K, Faullant R (2011) Why co-creation experience matters? Creative experience and its impact on the quantity and quality of creative contributions. R & D Management 41: 259–273.

[79] Jin B, Park JY, Kim H-S (2010) What makes online community members commit? A social exchange perspective. pp. 587–599.

[80] Jeppesen LB, Frederiksen L (2006) Why do users contribute to firm-hosted user communities? The case of computer-controlled music instruments. Organization science 17: 45–63.

[81] Kosonen M, Gan C, Olander H, Blomqvist K (2013) My idea is our idea! Supporting user-driven innovation activities in crowdsourcing communities. International Journal of Innovation Management 17: 1–18.

[82] Schweitzer FM, Buchinger W, Gassmann O, Obrist M (2012) Crowdsourcing. Leveraging Innovation through Online Idea Competitions. Research Technology Management 55: 32–38.

[83] Battistella C, Nonino F (2012) Open innovation web-based platforms: The impact of different forms of motivation on collaboration. Innovation-Management Policy & Practice 14: 557–575.

[84] Blohm I, Bretschneider U, Leimeister JM, Krcmar H (2011) Does collaboration among participants lead to better ideas in IT-based idea competitions? An empirical investigation. International Journal of Networking & Virtual Organisations 9: 106–122.

[85] Battistella C, Nonino F (2013) Exploring the impact of motivations on the attraction of innovation roles in open innovation web-based platforms. Production Planning & Control 24: 226–245.

[86] Hess J, Randall D, Pipek V, Wulf V (2013) Involving users in the wild—Participatory product development in and with online communities. International Journal of Human-Computer Studies 71: 570–589.

[87] Lerner J, Pathak PA, Tirole J (2006) The Dynamics of Open-Source Contributors. The American Economic Review 96: 114–118.

[88] Balka K, Raasch C, Herstatt C (2014) The Effect of Selective Openness on Value Creation in User Innovation Communities. Journal of Product Innovation Management 31: 392–407.

[89] Ogawa S, Pongtanalert K (2013) Exploring Characteristics and Motives of Consumer Innovators: Community Innovators vs. Independent Innovators. Research Technology Management 56: 41–48.

[90] Yuxiang CZ, Qinghua Z (2014) Effects of extrinsic and intrinsic motivation on participation in crowdsourcing contest. Online Information Review 38: 896.

[91] Kuo-Ming C, Hui-Chun C (2009) Community based innovation: its antecedents and its impact on innovation success. Internet Research 19: 496–516.

[92] Jespersen KR (2011) Online channels and innovation: Are users being empowered and involved? International Journal of Innovation Management 15: 1141.

[93] Djelassi S, Decoopman I (2013) Customers' participation in product development through crowdsourcing: Issues and implications. Industrial Marketing Management 42: 683–692.

[94] Müller-Seitz G, Reger G (2010) Networking beyond the software code? an explorative examination of the development of an open source car project. Technovation 30: 627–634.

[95] Kosonen M, Gan C, Vanhala M, Blomqvist K (2014) User motivation and knowledge sharing in idea corwdsourcing. International Journal of Innovation Management 18: -1.

[96] Leimeister JM, Huber M, Bretschneider U, Krcmar H (2009) Leveraging Crowdsourcing: Activation-Supporting Components for IT-Based Ideas Competition. Journal of Management Information Systems 26: 197–224.

[97] Weiber R, Muhlhaus D, Kim JS, Hyun JH (2014) Motives, success factors, and planned activities in a community of innovation as a critical mass system. Total Quality Management & Business Excellence 25: 1105–1125.

[98] Füller J, Mühlbacher H, Matzler K, Jawecki G (2009) Consumer Empowerment Through Internet-Based Co-creation. Journal of Management Information Systems 26: 71–102.

[99] Nambisan S, Baron RA (2010) Different Roles, Different Strokes: Organizing Virtual Customer Environments to Promote Two Types of Customer Contributions. Organization Science 21: 554–572.

[100] Franke N, Keinz P, Klausberger K (2013) "Does This Sound Like a Fair Deal?": Antecedents and Consequences of Fairness Expectations in the Individual's Decision to Participate in Firm Innovation. Organization Science 24: 1495–1516.

[101] Dahlander L, Piezunka H (2014) Open to suggestions: How organizations elicit suggestions through proactive and reactive attention. Research Policy 43: 812–827.

[102] MacCormack A, Murray F, Wagner E (2013) Spurring Innovation Through Competitions. MIT Sloan Management Review 55: 25–32.

[103] Zhang C, Hahn J, De P (2013) Continued Participation in Online Innovation Communities: Does Community Response Matter Equally for Everyone? Information Systems Research 24: 1112–1130.

[104] Malhotra A, Majchrzak A (2014) Managing Crowds in Innovation Challenges. California Management Review 56: 103–123.

[105] Roberts D, Hughes M, Kertbo K (2014) Exploring consumers' motivations to engage in innovation through co-creation activities. European Journal of Marketing 48: 147–169.

[106] Martinez-Torres MR (2013) Application of evolutionary computation techniques for the identification of innovators in open innovation communities. Expert Systems with Applications 40: 2503–2510.

[107] Stewart D (2005) Social status in an open-source community. American Sociological Review 70: 823–842.

[108] Fleming L, Waguespack DM (2007) Brokerage, boundary spanning, and leadership in open innovation communities. Organization Science 18: 165–180.

[109] Füller J, Hutter K, Hautz J, Matzler K (2014) User roles and contributions in innovation-contest communities. Journal of Management Information Systems 31: 273–308.

[110] Martinez-Torres MR, Toral SL, Barrero F, Cortes F (2010) The role of Internet in the development of future software projects. Internet Research 20: 72–86.

[111] Martins CS, Patricio L (2013) Understanding participation in company social networks. Journal of Service Management 24: 567–587.

[112] Jespersen KR (2010) User-Involvement and open Innovation: The Case of Decision-maker Openness. International Journal of Innovation Management 14: 471–489.

[113] Dahlander L, Frederiksen L (2012) The core and cosmopolitans: A relational view of innovation in user communities. Organization science 23: 988–1007.

[114] Sigala M (2012) Social networks and customer involvement in new service development (NSD). International Journal of Contemporary Hospitality Management 24: 966–990.

[115] Lampel J, Jha PP, Bhalla A (2012) Test-Driving the Future: How Design Competitions Are Changing Innovation. Academy of Management Perspectives 26: 71–85.

[116] Koch S (2004) Profiling an Open Source Project Ecology and Its Programmers. Electronic Markets 14: 77–88.

[117] Xu B, Lin Z, Xu Y (2011) A Study of Open Source Software Development from Control Perspective. Journal of Database Management 22: 26.

[118] Fagerholm F, Sanchez Guinea A, Borenstein J, Munch J (2014) Onboarding in Open Source Projects. IEEE Software 31: 54–61.

[119] Levine SS, Prietula MJ (2014) Open Collaboration for Innovation: Principles and Performance. Organization Science 25: 1414–1433.

[120] Lee H, Han J, Suh Y (2014) Gift or threat? An examination of voice of the customer: The case of MyStarbucksIdea.com. Electronic Commerce Research and Applications 13: 205–219.

[121] Parjanen S, Hennala L, Konsti-Laakso S (2012) Brokerage functions in a virtual idea generation platform: Possibilities for collective creativity? Innovation-Management Policy & Practice 14: 363–374.

[122] Villarroel JA, Taylor JE, Tucci CL (2013) Innovation and learning performance implications of free revealing and knowledge brokering in competing communities: insights from the Netflix Prize challenge. Computational and Mathematical Organization Theory 19: 42–77.

[123] Hutter K, Hautz J, Fueller J, Mueller J, Matzler K (2011) Communitition: The Tension between Competition and Collaboration in Community-Based Design Contests. Creativity and Innovation Management 20: 3–21.

[124] Cheng J (2008) How Macromedia Used Blogs to Build Its Developers' Communities: A Case Study. Performance Improvement Quarterly 21: 43–58.

[125] Mallapragada G, Grewal R, Lilien G (2012) User-Generated Open Source Products: Founder's Social Capital and Time to Product Release. Marketing Science 31: 474–492.

[126] Singh PV (2010) The Small-World Effect: The Influence of Macro-Level Properties of Developer Collaboration Networks on Open-Source Project Success. Acm Transactions on Software Engineering and Methodology 20.

[127] Barcellini F, Detienne F, Burkhardt J-M (2009) Participation in online interaction spaces: Design-use mediation in an Open Source Software community. International Journal of Industrial Ergonomics 39: 533–540.

[128] Alavi S, Ahuja V, Medury Y (2012) Metcalfe's law and operational, analytical and collaborative CRM-using online business communities for co-creation. Journal of Targeting, Measurement & Analysis for Marketing 20: 35–45.

[129] Boudreau KJ (2012) Let a Thousand Flowers Bloom? An Early Look at Large Numbers of Software App Developers and Patterns of Innovation. Organization Science 23: 1409–1427.

[130] Paulini M, Murty P, Maher ML (2013) Design processes in collective innovation communities: a study of communication. Codesign-International Journal of Cocreation in Design and the Arts 9: 90–112.

[131] Setia P, Rajagopalan B, Sambamurthy V, Calantone R (2012) How Peripheral Developers Contribute to Open-Source Software Development. Information Systems Research 23: 144–163.

[132] Martinez-Torres MD (2014) Analysis of activity in open-source communities using social network analysis techniques. Asian Journal of Technology Innovation 22: 114–130.

[133] Son J, Amrut S, Manchiraju S, Fiore AM, Niehm LS (2012) Consumer adoption of online collaborative customer co-design. Journal of Research in Interactive Marketing 6: 180–197.

[134] Liu H, Zhang J, Liu R, Li G (2014) A model for consumer knowledge contribution behavior: the roles of host firm management practices, technology effectiveness, and social capital. Information Technology and Management 15: 255–270.

[135] Juell-Skielse G, Hjalmarsson A, Juell-Skielse E, Johannesson P, Rudmark D (2014) Contests as innovation intermediaries in open data markets. Information Polity: The International Journal of Government & Democracy in the Information Age 19: 247–262.

[136] Henttonen K, Pussinen P, Koivumäki T (2012) Managerial Perspective on Open Source Collaboration and Networked Innovation. Journal of Technology Management & Innovation 7: 135–147.

[137] Stam W (2009) When does community participation enhance the performance of open source software companies? Research Policy 38: 1288–1299.

[138] Birkinshaw J, Bouquet C, Barsoux J-L (2011) The 5 Myths of Innovation. MIT Sloan Management Review 52: 43–50.

[139] Mount M, Martinez MG (2014) Social Media: A Tool for Open Innovation. California Management Review 56: 124–143.

[140] Rossi C (2011) Online consumer communities, collaborative learning and innovation. Measuring Business Excellence 15: 46–62.

[141] Mount M, Marian Garcia M (2014) Social Media: A Tool for Open Innovation. California Management Review 56: 124–143.

[142] Abdelkafi N, Blecker T, Raasch C (2009) From open source in the digital to the physical world: a smooth transfer? Management Decision 47: 1610–1632.

[143] Franke N, Hader C (2014) Mass or Only 'Niche Customization'? Why We Should Interpret Configuration Toolkits as Learning Instruments. Journal of Product Innovation Management 31: 1214–1234.

[144] Kohler T, Matzler K, Fueller J (2009) Avatar-based innovation: Using virtual worlds for real-world innovation. Technovation 29: 395–407.

[145] Ryzhkova N (2012) Web-based customer innovation: A replication with extension. Innovation: Management, Policy & Practice 14: 416–430.

[146] Ghazawneh A (2011) The power of platforms for software development in open innovation networks. International Journal of Networking & Virtual Organisations 9: 140–154.

[147] Dahlander L (2007) Penguin in a new suit: a tale of how de novo entrants emerged to harness free and open source software communities. Industrial and corporate change 16: 913–943.

[148] Guimarães ALS, Korn HJ, Shin N, Eisner AB (2013) The Life Cycle of Open Source Software Development Communities. Journal of Electronic Commerce Research 14: 167–182.

[149] Bianchi AJ, Kang SM, Stewart D (2012) The Organizational Selection of Status Characteristics: Status Evaluations in an Open Source Community. Organization Science 23: 341–354.

[150] Saldanha FP, Cohendet P, Pozzebon M (2014) Challenging the Stage-Gate Model in Crowdsourcing: The Case of Fiat Mio in Brazil. Technology Innovation Management Review 4: 28–35.

[151] Wei W (2013) An empirically derived framework of web-based interactive innovation practices. Innovation-Management Policy & Practice 15: 69–82.

[152] Antorini YM, Muñiz JAM, Askildsen T (2012) Collaborating With Customer Communities: Lessons from the Lego Group. MIT Sloan Management Review 53: 73–95.

[153] Jarvenpaa SL, Tuunainen VK (2013) How Finnair Socialized Customers for Service Co-Creation with Social Media. Mis Quarterly Executive 12: 125–136.

[154] di Gangi PM, Wasko MM, Hooker RE (2010) Getting customers' ideas to work for you: Learning from Dell how to succeed with online user innovation communities. Mis Quarterly Executive 9: 213–228.

[155] Huang Y, Singh PV, Srinivasan K (2014) Crowdsourcing New Product Ideas Under Consumer Learning. Management science 60: 2138–2159.

[156] Schlagwein D, Bjorn-Andersen N (2014) Organizational Learning with Crowdsourcing: The Revelatory Case of LEGO. Journal of the Association for Information Systems 15: 754–778.

[157] Nambisan S, Nambisan P (2008) How to Profit From a Better 'Virtual Customer Environment'. MIT Sloan Management Review 49: 53–61.

[158] King A, Lakhani KR (2013) Using Open Innovation to Identify the Best Ideas. MIT Sloan Management Review 55: 41–48. PMC5739322 29276450

[159] West J, O'Mahony S (2008) The Role of Participation Architecture in Growing Sponsored Open Source Communities. Industry and innovation 15: 145–168.

[160] Williams RL, Cothrel J (2000) Four Smart Ways to Run Online Communities. Sloan management review 41: 81–91.

[161] Saxton GD, Oh O, Kishore R (2013) Rules of Crowdsourcing: Models, Issues, and Systems of Control. Information Systems Management 30: 2–20.

[162] Teigland R, Di Gangi PM, Flaten BT, Giovacchini E, Pastorino N (2014) Balancing on a tightrope: Managing the boundaries of a firm-sponsored OSS community and its impact on innovation and absorptive capacity. Information and Organization 24: 25–47.

[163] Ogawa S, Piller FT (2006) Reducing the Risks of New Product Development. MIT Sloan Management Review 47: 65.

[164] Latusek-Jurczak D, Prystupa-Rządca K (2014) Collaboration and trust-building in open innovation community. Journal of Economics & Management: 47–62.

[165] Filieri R (2013) Consumer co-creation and new product development: A case study in the food industry. Marketing Intelligence & Planning 31: 40–53.

[166] Riedl C, Blohm I, Leimeister JM, Krcmar H (2013) The Effect of Rating Scales on Decision Quality and User Attitudes in Online Innovation Communities. International Journal of Electronic Commerce 17: 7–36.

[167] Bothos E, Apostolou D, Gregoris M (2009) Collective intelligence for idea management with Internet-based information aggregation markets. Internet Research 19: 26–41.

[168] Toubia O, Laurent F (2007) Adaptive Idea Screening Using Consumers. Marketing Science 26: 342–360,448,446.

[169] Yücesan E (2013) An efficient ranking and selection approach to boost the effectiveness of innovation contests. Iie Transactions 45: 751–762.

[170] Poetz MK, Schreier M (2012) The Value of Crowdsourcing: Can Users Really Compete with Professionals in Generating New Product Ideas? Journal of Product Innovation Management 29: 245–256.

[171] Piller FT, Walcher D (2006) Toolkits for idea competitions: a novel method to integrate users in new product development. R&d Management 36: 307–318.

[172] Cheng CCJ, Tsai H-T, Krumwiede D (2013) How to enhance new product creativity in the online brand community? Innovation: Management, Policy & Practice 15: 83–96.

[173] Zheng H, Xie Z, Hou W, Li D (2014) Antecedents of solution quality in crowdsourcing: The sponsor's perspective. Journal of Electronic Commerce Research 15: 212–224.

[174] Scupola A, Nicolajsen HW (2013) Using Social Media for Service Innovations: Challenges and Pitfalls. International Journal of E-Business Research 9: 27.

[175] Terwiesch C Jr., Xu Y (2008) Innovation contests, open innovation, and multiagent problem solving. Management science 54: 1529–1543.

[176] Roberts DL, Candi M (2014) Leveraging Social Network Sites in New Product Development: Opportunity or Hype? Journal of Product Innovation Management 31: 105–117.

[177] Mortara L, Ford SJ, Jaeger M (2013) Idea Competitions under scrutiny: Acquisition, intelligence or public relations mechanism? Technological Forecasting and Social Change 80: 1563–1578.

[178] Sarkar S, Costa AIA (2008) Dynamics of open innovation in the food industry. Trends in Food Science & Technology 19: 574–580.

[179] Colombo MG, Piva E, Rossi-Lamastra C (2014) Open innovation and within-industry diversification in small and medium enterprises: The case of open source software firms. Research Policy 43: 891–902.

[180] Kim JH, Bae Z-T, Kang SH (2008) The role of online brand community in new product development: Case studies on digital product manufacturers in Korea. International Journal of Innovation Management 12: 357–376.

[181] Roberts N, Grover V (2012) Leveraging Information Technology Infrastructure to Facilitate a Firm's Customer Agility and Competitive Activity: An Empirical Investigation. Journal of Management Information Systems 28: 231–270.

[182] van Dijk J, Antonides G, Schillewaert N (2014) Effects of co-creation claim on consumer brand perceptions and behavioural intentions. International Journal of Consumer Studies 38: 110–118.

[183] Huizingh EKRE (2011) Open innovation: State of the art and future perspectives. Technovation 31: 2–9.

[184] Ulkuniemi P, Pekkarinen S, Westh Nicolajsen H, Scupola A (2011) Investigating issues and challenges for customer involvement in business services innovation. Journal of Business & Industrial Marketing 26: 368–376.

[185] Wallin MW, Von Krogh G (2010) Organizing for Open Innovation:: Focus on the Integration of Knowledge. Organizational dynamics 39: 145–154.

[186] Alam I, Perry C (2002) A customer-oriented new service development process. Journal of services Marketing 16: 515–534.

[187] Raasch C (2011) Product development in open design communities: A process perspective. International Journal of Innovation & Technology Management 8: 557–575.

[188] Reinhardt R, Bullinger AC, Gurtner S (2015) Open Innovation in Health Care Challenges and Opportunities in Health Care Management: Springer pp. 237–246.

[189] Martini A, Massa S, Testa S (2013) The firm, the platform and the customer: A “double mangle” interpretation of social media for innovation. Information & Organization 23: 198–213.

[190] Tuckermann H. Multirational Management in Hospitals In: Schedler K, Rüegg-Stürm J, editors. Multi-rational Management: Mastering Conflicting Demands in a Pluralistic Environment. Palgrave Macmillan; 2014 71–90.

[191] Guenduez AA, Schedler K (2014) Challenges for Public Management in Multirational Public Organisations. International Public Management Review 15: 58–76.

[192] Patel V, Abramson EL, Edwards A, Malhotra S, Kaushal R (2011) Physicians’ potential use and preferences related to health information exchange. International Journal of Medical Informatics 80: 171–180. 10.1016/j.ijmedinf.2010.11.008 21156351

[193] Reay T, Hinings CR (2009) Managing the rivalry of competing institutional logics. Organization studies 30: 629–652.

[194] Rubinelli S, Schulz PJ, Nakamoto K (2009) Health literacy beyond knowledge and behaviour: letting the patient be a patient. International Journal of Public Health 54: 307–311. 10.1007/s00038-009-0052-8 19641846

[195] Henwood F, Wyatt S, Hart A, Smith J (2003) ‘Ignorance is bliss sometimes’: constraints on the emergence of the ‘informed patient’in the changing landscapes of health information. Sociology of Health & Illness 25: 589–607. 12919447 10.1111/1467-9566.00360

[196] Thorne SE, Ternulf Nyhlin K, Paterson BL (2000) Attitudes toward patient expertise in chronic illness. International journal of nursing studies 37: 303–311. 10760537 10.1016/s0020-7489(00)00007-9

[197] Christensen M, Hewitt-Taylor J (2006) Empowerment in nursing: paternalism or maternalism? British Journal of Nursing 15: 695–699. 16926716 10.12968/bjon.2006.15.13.21478

[198] Coulter A, Ellins J (2007) Effectiveness of strategies for informing, educating, and involving patients. BMJ 335: 24 17615222 10.1136/bmj.39246.581169.80 PMC1910640

[199] Kuenne CW, Temidayo A, Moeslein KM. Online Innovation Intermediaries In Healthcare. 2013; paper 186. Available: http://aisel.aisnet.org/ecis2013_cr/186.

[200] Ferrand E, Lemaire F, Regnier B, Kuteifan K, Badet M, Asfar P, et al (2003) Discrepancies between perceptions by physicians and nursing staff of intensive care unit end-of-life decisions. American journal of respiratory and critical care medicine 167: 1310–1315. 12738597 10.1164/rccm.200207-752OC

[201] Mulley AG, Trimble C, Elwyn G (2012) Stop the silent misdiagnosis: patients’ preferences matter. Bmj 345: e6572 10.1136/bmj.e6572 23137819

[202] Zanini C, Sarzi-Puttini P, Atzeni F, Di Franco M, Rubinelli S (2015) Building bridges between doctors and patients: the design and pilot evaluation of a training session in argumentation for chronic pain experts. BMC medical education 15: 1. 25986603 10.1186/s12909-015-0374-6 PMC4469318

[203] Wearden A, Peters S (2008) Therapeutic techniques for interventions based on Leventhal's common sense model. British Journal of Health Psychology 13: 189–193. 10.1348/135910708X295613 18492318

[204] Heyduck K, Meffert C, Glattacker M (2014) Illness and treatment perceptions of patients with chronic low back pain: characteristics and relation to individual, disease and interaction variables. Journal of clinical psychology in medical settings 21: 267–281. 10.1007/s10880-014-9405-4 25100026

[205] Malone M, Harris R, Hooker R, Tucker T, Tanna N, Honnor S (2004) Health and the Internet—changing boundaries in primary care. Family practice 21: 189–191. 15020390 10.1093/fampra/cmh215

[206] Broom A (2005) Virtually he@lthy: the impact of internet use on disease experience and the doctor-patient relationship. Qualitative Health Research 15: 325–345. 15761103 10.1177/1049732304272916

[207] Wald HS, Dube CE, Anthony DC (2007) Untangling the Web—The impact of Internet use on health care and the physician—patient relationship. Patient Education and Counseling 68: 218–224. 17920226 10.1016/j.pec.2007.05.016

[208] Rosenberg W, Donald A (1995) Evidence based medicine: an approach to clinical problem-solving. BMJ: British Medical Journal 310: 1122 7742682 10.1136/bmj.310.6987.1122 PMC2549505

[209] Schulz PJ, Nakamoto K (2013) Health literacy and patient empowerment in health communication: the importance of separating conjoined twins. Patient education and counseling 90: 4–11. 10.1016/j.pec.2012.09.006 23063359

[210] Lavis J, Catallo C (2013) Bridging the worlds of research and policy in European health systems. Copenhagen, Denmark: WHO Regional Office for Europe 4: 5. 28981248

[211] Lavis JN, McCutchen B, Bopardikar A. Issue Brief: Engaging Civil Society in Supporting Research Use in Health Systems. Hamilton, Canada: McMaster Health Forum, 23 11 2009 Available: https://macsphere.mcmaster.ca/bitstream/11375/14865/1/fulltext.pdf.

[212] Weitzman ER, Kaci L, Mandl KD (2010) Sharing medical data for health research: the early personal health record experience. Journal of Medical Internet Research 12: e14 10.2196/jmir.1356 20501431 PMC2956225

[213] Wicks P, Massagli M, Frost J, Brownstein C, Okun S, Vaughan T, et al (2010) Sharing health data for better outcomes on PatientsLikeMe. Journal of medical Internet research 12: e19 10.2196/jmir.1549 20542858 PMC2956230

[214] Ferguson T (2000) Online patient-helpers and physicians working together: a new partnership for high quality health care. BMJ: British Medical Journal 321: 1129 11061737 10.1136/bmj.321.7269.1129 PMC1118902

[215] Hartmann M, Prinz A, Leimeister JM. Open Innovation im Healthcare-Systematische Entwicklung von Ideenwettbewerben am Beispiel von Patienten mit amyotropher Lateralsklerose; 2010. pp. 102–107. Available: http://cs.emis.de/LNI/Proceedings/Proceedings175/102.pdf

[216] Lasker JN, Sogolow ED, Sharim RR (2005) The role of an online community for people with a rare disease: content analysis of messages posted on a primary biliary cirrhosis mailinglist. Journal of medical Internet research 7: e10 15829472 10.2196/jmir.7.1.e10 PMC1550634

[217] Armstrong N, Koteyko N, Powell J (2012) ‘Oh dear, should I really be saying that on here?’: Issues of identity and authority in an online diabetes community. Health: 16: 347–365. 10.1177/1363459311425514 22067915

[218] Zrebiec J, Jacobson A (2001) What attracts patients with diabetes to an internet support group? A 21‐month longitudinal website study. Diabetic medicine 18: 154–158. 11251681 10.1046/j.1464-5491.2001.00443.x

[219] Househ M, Borycki E, Kushniruk A (2014) Empowering patients through social media: the benefits and challenges. Health Informatics Journal 20: 50–58. 10.1177/1460458213476969 24550564

[220] Magnezi R, Grosberg D, Novikov I, Ziv A, Shani M, Freedman LS (2015) Characteristics of patients seeking health information online via social health networks versus general Internet sites: a comparative study. Informatics for Health and Social Care 40: 125–138. 10.3109/17538157.2013.879147 24475937

[221] Wallace DJ, Gotto J. Hypothesis: bipolar illness with complaints of chronic musculoskeletal pain is a form of pseudofibromyalgia 2008 Elsevier. 256–259 10.1016/j.semarthrit.2007.04.008 17570468

[222] Edwards T (2008) Complementary Therapies for Genitourinary Pain Genitourinary Pain And Inflammation: Springer 325–334.

[223] Edwards SJL, Braunholtz DA (2000) Can unequal be more fair? A response to Andrew Avins. Journal of Medical Ethics 26: 179–182. 10860209 10.1136/jme.26.3.179 PMC1733212

[224] Morahan-Martin JM (2004) How internet users find, evaluate, and use online health information: a cross-cultural review. CyberPsychology & Behavior 7: 497–510. 15667044 10.1089/cpb.2004.7.497

[225] Eysenbach G, Köhler C (2002) How do consumers search for and appraise health information on the world wide web? Qualitative study using focus groups, usability tests, and in-depth interviews. Bmj 324: 573–577. 11884321 10.1136/bmj.324.7337.573 PMC78994

[226] Demiris G (2006) The diffusion of virtual communities in health care: concepts and challenges. Patient education and counseling 62: 178–188. 16406472 10.1016/j.pec.2005.10.003

[227] Hajli MN, Sims J, Featherman M, Love PED (2014) Credibility of information in online communities. Journal of Strategic Marketing: 1–16.

[228] Adams SA (2010) Revisiting the online health information reliability debate in the wake of “web 2.0”: an inter-disciplinary literature and website review. International journal of medical informatics 79: 391–400. 10.1016/j.ijmedinf.2010.01.006 20188623

[229] Schumacher KR, Stringer KA, Donohue JE, Yu S, Shaver A, Caruthers RL, et al (2014) Social media methods for studying rare diseases. Pediatrics 133: e1345–e1353. 10.1542/peds.2013-2966 24733869 PMC4006435

[230] Grabher G, Ibert O, Flohr S (2008) The Neglected King: The Customer in the New Knowledge Ecology of Innovation. Economic Geography 84: 253–280.

[231] Eysenbach G, Powell J, Kuss O, Sa E-R (2002) Empirical studies assessing the quality of health information for consumers on the world wide web: a systematic review. Jama 287: 2691–2700. 12020305 10.1001/jama.287.20.2691

[232] Gagliardi A, Jadad AR (2002) Examination of instruments used to rate quality of health information on the internet: chronicle of a voyage with an unclear destination. Bmj 324: 569–573. 11884320 10.1136/bmj.324.7337.569 PMC78993

[233] Kaicker J, Debono VB, Dang W, Buckley N, Thabane L (2010) Assessment of the quality and variability of health information on chronic pain websites using the DISCERN instrument. BMC medicine 8: 59 10.1186/1741-7015-8-59 20939875 PMC2967493

[234] Schroeder EB, Desai J, Schmittdiel JA, Paolino AR, Schneider JL, Goodrich GK, et al (2015) An Innovative Approach to Informing Research: Gathering Perspectives on Diabetes Care Challenges From an Online Patient Community. Interactive journal of medical research 4: e13 10.2196/ijmr.3856 26126421 PMC4526969

