# Supplementary material for: What Online User Innovation Communities Can Teach Us about Capturing the Experiences of Patients Living with Chronic Health Conditions. A Scoping Review
Source: PLoS One. 2016 Jun 7;11(6):e0156175. doi: 10.1371/journal.pone.0156175 (PMC4896620; doi:10.1371/journal.pone.0156175)
Supplement: S1 Protocol — (PDF) [file pone.0156175.s002.pdf]

Scoping review methodology

Despite their similarities, the scoping review methodology distinguishes itself from the traditional systematic review approach in terms of its nature and purpose as outlined by Brien and colleagues [1] (see Table 1). The scoping review allows the researcher to map the existing literature on a certain topic in a systematic and comprehensive way. The findings of the scoping review may then in turn provide the ideal basis for a systematic literature review.

Given the interdisciplinary nature and purpose of this study, we chose to follow the five-stage framework of the scoping review methodology as proposed by Arksey and O'Malley [2]. Aiming to link the available evidence from various disciplines to the healthcare context our research question was broad in nature, which in turn required post hoc adjustments of the initial inclusion and exclusion criteria. Further, as we were aiming to map the field study quality was not prioritized.

Table 1. Comparison of the characteristics of scoping and systematic reviews [1].

Systematic Review	Scoping Review
Focused research question with narrow parameters	Research question(s) often broad
Inclusion/exclusion usually defined at outset	Inclusion/exclusion can be developed post hoc
Quality filters often applied	Quality not an initial priority
Detailed data extraction	May or may not involve data extraction
Quantitative synthesis often performed	Synthesis more qualitative, and typically not quantitative
Formally assesses the quality of studies and generates a conclusion relating to the focused research question	Used to identify parameters and gaps in a body of literature

Research Protocol

following Arksey and O'Malley [2]. The research questions were developed and refined in a collaborative effort of the research team (JA, CZ, SR).

1. Identifying the Research Question

The specific questions guiding this scoping review were:

(1) In which contexts are online open innovation communities (OOICs) used and how are they initiated? (firm vs user initiated)
(2) How are OOICs organized and structured?
(3) Why do users contribute to OOICs?
(4) What are the challenges when involving OOICs in the innovation process?
(5) What do we know about managing OOICs?
(6) How are ideas generated by OOICs being selected?
(7) Are OOICs a successful idea acquisition mechanism?
(8) What are the measures of success for OOICs?

Thus, the purposes of this scoping review will be to

1. Map the literature on OOICs in order to identify topics of interest relevant for the healthcare setting
2. Identify the key principles ("lessons learned") of OOICs
3. Determine whether OOICs constitute a promising approach for healthcare by taking the particularities of the setting into account

2. Study Identification

The search strategy was developed by the research team (JA, CZ, SR). In an effort to balance feasibility with comprehensiveness of the scoping process, we decided to focus on scientific articles published in English. Publications in other languages as well as grey literature were important sources that informed data extraction and analysis.

It should also be noted that while theoretical and conceptual work served as a foundation and important guidance to the research process and interpretation of the findings, only empirical studies are reported in the findings of the scoping review.

Due to the heterogeneity of the field in terms of disciplines and terminology, our search strategy might have failed to capture some relevant studies. We tried to minimize this risk by conducting a preliminary review of the literature to inform both our search strategy as well as study selection criteria.

Keywords:

"open innovation" OR "idea challenge" OR "crowd sourcing" OR "crowd-sourcing" OR (innovation AND "internet user") OR (innovation AND ("social media" OR facebook OR twitter)) OR "user co-creation" OR "open innovation community" or "internet community" OR "web-based community" OR "online community" OR "online innovation community" 1995-2014, English

Databases searched to import citation and abstract into EndNote

- ISI Web of Science
- Communication & Mass Media Complete
- psycINFO
- Business Source Premier
- MEDLINE
- ABI Inform Global
- ABI Trade & Industry

After elimination of incomplete files and duplicates, the remaining files were converted to RIS format and were imported into EPPI reviewer 4, a web-based software tool for research synthesis.

3. Study selection

The research team (JA, CZ, SR) engaged in an iterative process to refine the inclusion and exclusion criteria. Inter-coder reliability was assessed for 20% of the records and yielded 90% correspondence.

Final inclusion criteria:

- 1995-2014, English, empirical study
- Focus: autonomous or firm-sponsored/hosted online community with end-user (consumer) activities focused on co-creation/innovation (e.g. product improvement)

Studies were excluded if they examined online communities that did not involve user innovation activities (e.g. dating communities) or that did not focus on end-user (consumer involvement) but rather employee or supplier integration. Studies reporting on offline initiatives to integrate consumers in the innovation process, studies investigating innovation intermediaries or crowdfunding practices were also excluded.

4. Charting the Data

Two researchers (JA, CZ) developed and refined the data charting form (Table 2). Data extraction was performed by JA.

Articles Characteristics	Study Characteristics	Study Findings
Title	Field of Research	Results
Year of publication	Research Focus	Conclusion
Author(s)	Study Design	
Journal	Methods	
	Research Questions	
	Hypothesis	

5. Collating, Summarizing, and Reporting Results

- Mapping and categorization of areas of research (study focus / research questions)
- Narrative account of the evidence
- Identification of "lessons learned" (best practice approaches)

6. Consulting Knowledge Users

Due to resource constraints, the optional consultation with knowledge users was not conducted. This is however planned for a forthcoming project to be initiated in 2016.

References

1. Brien SE, Lorenzetti DL, Lewis S, Kennedy J, Ghali WA (2010) Overview of a formal scoping review on health system report cards. Implement Sci 5: 2.
2. Arksey H, O'Malley L (2005) Scoping studies: towards a methodological framework. International journal of social research methodology 8: 19-32.
