# Supplementary material for: What Online User Innovation Communities Can Teach Us about Capturing the Experiences of Patients Living with Chronic Health Conditions. A Scoping Review
Source: PLoS One. 2016 Jun 7;11(6):e0156175. doi: 10.1371/journal.pone.0156175 (PMC4896620; doi:10.1371/journal.pone.0156175)
Supplement: S1 Table — (PDF) [file pone.0156175.s003.pdf]

	Authors, Date	Research Focus	Category			
			User and community Factors	Organiza-tional Factors	Output and Outcome	Other
**1**	Abdelkafi, Blecker 2009	Transferability of OSS principles: applicability of the open source concept in product development outside software		**x**		
**2**	Alavi, Ahuja 2012	Identification of co-creators through individual network value	**x**			
**3**	Antikainen, Mäkipää 2010	Motivations/facilitators for user collaboration	**x**			
**4**	Antorini, Muñiz 2012	Interaction firm-users		**x**	**x**	
**5**	Antorini and Muñiz 2013	Interaction firm-users (challenges & rewards)		**x**	**x**	
**6**	Balka, Raasch 2014	Effects of selective openness on user value creation	**x**			
**7**	Barcellini, Detienne 2009	User roles fostering design-use mediation during the OSS design process by measuring participation	**x**			
**8**	Battistella and Nonino 2012	Impact of platform drivers on users' motivations	**x**			
**9**	Battistella and Nonino 2012	Motivations as function of different stages in the innova-tion process and the dependency on members	**x**			
**10**	Battistella and Nonino 2013	Effects of motivational systems and platforms models on the attraction of different innovation roles.	**x**			
**11**	Bayus 2013	Impact of individual’s past success in proposing imple-mented ideas on subsequent ideation efforts.	**x**		**x**	
**12**	Bianchi, Kang 2012	Status evaluations in OSS community (giving credit)	**x**			
**13**	Birkinshaw, Bouquet 2011	Myths in open innovation (company experiences)		**x**		
**14**	Blohm, Bretschneider 2011	Influence of collaboration on idea quality	**x**		**x**	
**15**	Bothos, Apostolou 2009	Tool development to support idea generation and eval-uation utilizing concept of information aggregation mar-kets (IAMs)		**x**		
**16**	Boudreau, Lacetera 2011	Impact of number of competitors on performance		**x**	**x**	
**17**	Boudreau 2012	Impact of number of contributors on performance			**x**	
**18**	Brabham 2010	Motivations for participating	**x**			
**19**	Capra, Francalanci 2008	Relationship among software design quality, develop-ment effort, and governance in OSS projects		**x**	**x**	
**20**	Cheng 2008	Strategy (Blogger)	**x**	**x**	**x**	
**21**	Cheng, Tsai 2013	Impact of online brand community on product creativity	**x**	**x**	**x**	
**22**	Chua and Banerjee 2013	Role of social media in supporting customer knowledge management		**x**		
**23**	Colombo, Piva 2014	Impact of firm involvement with OSS communities on		**x**	**x**	

	Authors, Date	Research Focus	Category			
			User and community Factors	Organiza-tional Factors	Output and Outcome	Other
		within-industry diversification				
**24**	Cromie and Ewing 2008	Description of research methodology to study OSS				x
**25**	Dahlander and Piezunka 2014	Strategies to elicit user contributions	x	x		
**26**	Dahlander 2007	Strategies to harness user community		x		
**27**	Dahlander and Frederiksen 2012	Effect of user network position on innovation	x		x	
**28**	de Toni, Biotto 2012	Identification of organizational design drivers that ena-ble self-organization		x		
**29**	di Gangi and Wasko 2009	Identification of factors influencing an organization's adoption decision		x	x	
**30**	di Gangi, Wasko 2010	Challenges of OIC		x		
**31**	Djelassi and Decoopman 2013	Effect of customer participation in product development on business model		x		
**32**	Evans-Cowley and Hollander 2010	Added value of web-based technologies for citizen participation			x	
**33**	Fagerholm, Sanchez Guinea 2014	Impact of mentoring on newcomer performance in OSS projects	x		x	
**34**	Filieri 2013	Analysis of innovation outputs when involving customers at the “fuzzy front end” of new product development		x	x	
**35**	Fleming and Waguespack 2007	Identification of community leaders within OIC	x			
**36**	Franke, Keinz 2013	Role of fairness expectations in the initial decision to contribute to a crowdsourcing system	x			
**37**	Franke and Hader 2014	Role of toolkit for user preference insight	x			
**38**	Füller, Jawecki 2007	Activities in an online consumer community<br>- the quality and quantity of ideas<br>- motivations to jointly innovate<br>- process through which innovations emerge<br>- users' willingness to share with inquiring companies	x		x	
**39**	Füller, Hutter 2011	Impact of co-creation experience on quantity and quality of creative contributions and further interest to participate in design competition	x		x	
**40**	Füller, Hutter 2014	Impact of user roles on quality and types of contribu-tions, and user interaction	x		x	
**41**	Füller, Matzler 2012	- Impact of personal user characteristics in the different stages of NPD	x		x	

	Authors, Date	Research Focus	Category			
			User and community Factors	Organiza-tional Factors	Output and Outcome	Other
		- Impact of creativity components on the users' intention to participate in co-creation projects				
**42**	Füller, Mühlbacher 2009	Impact of co-creation tool and tool support on<br>- ability of consumers to contribute to NPD<br>- perceived empowerment and enjoyment of the task<br>- intention to participate in future NPD projects	**x**	**x**		
**43**	Ghazawneh 2011	Use of product platform concept in open innovation networks (Strategies)		**x**		
**44**	Grabher and Ibert 2014	Virtually mediated collaboration and its specific contri-butions to knowledge creation	**x**			
**45**	Guimarães, Korn 2013	Development patterns of OSS projects in terms of effec-tiveness and activity levels	**x**		**x**	
**46**	Hann, Roberts 2013	Economic benefit for participants in OSS communities	**x**			
**47**	Hennala, Parjanen 2011	Development and testing of model for user involvement (ideation phase)	**x**	**x**	**x**	
**48**	Henttonen, Pussinen 2012	Managerial attitudes, expected benefits and key chal-lenges regarding involvement with OSS communities		**x**		
**49**	Hess, Randall 2013	Managing software development process	**x**	**x**		
**50**	Hildebrand, Häubl 2013	Effect of community feedback on product designs and their satisfaction with these self-designed products	**x**		**x**	
**51**	Huang, Singh 2014	Dynamics of user participation behavior	**x**	**x**	**x**	
**52**	Hutter, Hautz 2011	Co-operation and competition behavior in community-based idea and design contests	**x**		**x**	
**53**	Jarvenpaa and Tuunainen 2013	Co-creation strategy by using socialization tactics		**x**		
**54**	Jeppesen and Frederiksen 2006	Identification of user attributes / motivations	**x**			
**55**	Jespersen 2010	Impact of decision-maker openness on user involvement	**x**	**x**		
**56**	Jespersen 2011	Role of online channels in empowerment and involve-ment of different user types at different stages of NPD process	**x**	**x**		
**57**	Jin, Park 2010	Impact of sociability and usability on user participation	**x**			
**58**	Juell-Skielse, Hjalmarsson 2014	Functions & design of innovation contests		**x**		
**59**	Khan, Ludlow 2014	Effectiveness of participatory information and communication technology (ICT) tools for urban planning		**x**	**x**	
**60**	Kim, Bae 2008	Utilization of online brand communities in new product	**x**	**x**		

	Authors, Date	Research Focus	Category			
			User and community Factors	Organiza-tional Factors	Output and Outcome	Other
		development (NPD)				
**61**	King and Lakhani 2013	Strategy options incl. key success factors and common problems		x		
**62**	Koch 2004	Assessment of OSS development paradigm: differences between OSS model and commercial organization of work		x		
**63**	Kohler, Matzler 2009	Opportunities of virtual worlds as tools for innovation		x		
**64**	Kosonen, Gan 2013	Impact of community trust and community support on users' intentions to share knowledge	x			
**65**	Kosonen, Gan 2014	Impact of propensity to trust, intrinsic motivation, and extrinsic motivation on intentions to share knowledge	x			
**66**	Kuo-Ming and Hui-Chun 2009	Factors promoting user participation in community based innovation	x			
**67**	Lampel, Jha 2012	Set up, management & design of design competitions		x		
**68**	Latusek-Jurczak and Prystupa-Rządca 2014	Trust development in OIC		x		
**69**	Latzko-Toth 2014	User as co-designers of socio-technical device	x			
**70**	Lee, Han 2014	Effects of customer expectations on user interactions and organizational innovation	x	x	x	
**71**	Lee, Hwang 2012	Open innovation practices in the public sector		x		
**72**	Leimeister, Huber 2009	Impact of incentives on user motivation perception	x	x		
**73**	Lerner, Pathak 2006	User contribution distribution to OSS projects	x	x		
**74**	Levine and Prietula 2014	Identification of principles of open collaboration for innovation and factors impacting its performance	x		x	
**75**	Liu, Zhang 2014	Effects of management practices, social capital, and technological factors on consumer knowledge contribution behavior	x	x		
**76**	Lüders, Folstad 2014	Role of social media in empowering citizens in politics.		x		
**77**	MacCormack, Murray 2013	Functioning of innovation contests		x		
**78**	Malhotra and Majchrzak 2014	Effect of knowledge integration guidelines, and incentives to use those guidelines on competitive advantage potential of solutions generated by the innovation challenge participants	x	x	x	
**79**	Mallapragada, Grewal 2012	Factors impacting time to product release	x		x	
**80**	Marchi, Giachetti 2011	Identification of innovative users	x			

	Authors, Date	Research Focus	Category			
			User and community Factors	Organiza-tional Factors	Output and Outcome	Other
**81**	Martinez-Torres 2014	Identification of main dimensions affecting user participation in OSS community	**x**			
**82**	Martinez-Torres 2013	Identification of user innovators	**x**			
**83**	Martinez-Torres 2014	- Correlation between different forms of participation - Identification of user innovators	**x**		**x**	
**84**	Martinez-Torres, Toral 2010	Analysis of OSS community structure	**x**			
**85**	Martini, Massa 2014	Co-creation strategy		**x**	**x**	
**86**	Martini, Massa 2013	Development stages of co-creation project		**x**		
**87**	Martins and Patricio 2013	Impact of goals to join and continuously participate in company social networks and perceived drivers of this participation	**x**			
**88**	Mortara, Ford 2013	Reasons for and effects of using innovation contests as an acquisition mechanism		**x**	**x**	
**89**	Mount and Martinez 2014	Motivations, implementation, impacts, and challenges of application of social media for open innovation		**x**		
**90**	Müller-Seitz and Reger 2010	Identification of parallels to OSS projects and the barriers regarding implementation of OSS mechanisms to devel-opment of a non-software related product		**x**		
**91**	Nambisan and Baron 2010	Factors shaping diverse customer contributions in online customer forums	**x**			
**92**	Nambisan and Nambisan 2008	Design of virtual consumer environment	**x**	**x**		
**93**	Nätti, Hurmelinna-Laukkanen 2014	Effects of network orchestration on absorptive capacity in service innovation communities	**x**	**x**		
**94**	Ogawa and Piller 2006	Strategies of collective customer commitment and their potential to avoid product failures		**x**		
**95**	Ogawa and Pongtanalert 2013	Comparison of motivations and behavior of solo vs. community innovators	**x**			
**96**	Parjanen, Hennala 2012	Impact of brokerage functions on collective creativity	**x**	**x**	**x**	
**97**	Paulini, Murty 2013	Design process visualisations of collective innovation	**x**		**x**	
**98**	Piller and Walcher 2006	Design, implementation and performance assessment of a toolkit for idea competitions as a method for NPD		**x**	**x**	
**99**	Poetz and Schreier 2012	Comaprison of quality of new product ideas generated by users through a crowdsourcing process compared with those generated by firm’s professionals			**x**	

	Authors, Date	Research Focus	Category			
			User and community Factors	Organiza-tional Factors	Output and Outcome	Other
**100**	Raasch 2011	Applicability of OSS model to tangible products: open design processes	x	x		
**101**	Raasch, Herstatt 2009	Generalization of the OSS model to non-industry-specific level		x		
**102**	Raasch and von Hippel 2013	Relative importance of user motivations to contribute to innovation projects	x			
**103**	Riedl, Blohm 2013	Effects of rating scales on user attitudes towards website and decision quality	x	x		
**104**	Roberts and Candi 2014	Effect of using social network sites in new product development process		x	x	
**105**	Roberts, Hughes 2014	Framework of user motivations across types of co-creation	x			
**106**	Roberts and Grover 2012	Impact of information technology on a firm's customer agility and competitive activity		x		
**107**	Rossi 2011	Managerial challenges when leveraging customers' creative talent		x	x	
**108**	Russo-Spena and Mele 2012	Innovation as a process of co-creation		x		
**109**	Ryzhkova 2012	Diffusion of web-based product innovation		x		
**110**	Saldanha, Cohendet 2014	Management of crowdsourcing project (model)	x	x	x	
**111**	Sarkar and Costa 2008	Effects of open innovation strategies on innovation capabilities and market outcomes		x	x	
**112**	Sasinovskaya and Anderson 2011	Added value of online community by combining design toolkits and social network features, and the perceived barriers for using them		x	x	
**113**	Saxton, Oh 2013	Deelopment of taxonomy of types of crowdsourcing models		x		
**114**	Schlagwein and Bjorn-Andersen 2014	Crowdsourcing as contributing to organizational learning		x		
**115**	Schweitzer, Buchinger 2012	Comparison of expense and results of online idea com-petitions with focus groups for idea generation			x	
**116**	Scupola and Hanne Westh 2010	Customer roles and involvment in the service innovation process	x	x		
**117**	Scupola and Nicolajsen 2013	Role of social software for user invovlement in idea generation process of service innovations		x	x	
**118**	Seltzer and Mahmoudi 2013	Applicability of crowdsourcing and open innovation in		x		

	Authors, Date	Research Focus	Category			
			User and community Factors	Organiza-tional Factors	Output and Outcome	Other
		planning				
**119**	Setia, Rajagopalan 2012	Conribution of peripheral developers to product quality and diffusion	x		x	
**120**	Shen and Monge 2011	Social drivers that shape the collaboration dynamics of an OSS community	x			
**121**	Sigala 2012	Customer contributions (role of contribut., ideation)	x			
**122**	Singh 2010	Role of customers' contributions in social networks for New service development	x	x	x	
**123**	Son, Amrut 2012	Perceived benefits and impediments offered by a collaborative co-design website and its influence on user acceptance of this technology	x			
**124**	Spaeth, Stuermer 2010	Enableing factors for a push model of open innova-tion		x		
**125**	Spiliotopoulou, Charalabidis 2014	Development of a framework for the application of crowdsourcing practices in the public sector		x		
**126**	Stam 2009	Influence of participation in OIC on the innovative and financial performance of firms commercializing OSS		x	x	
**127**	Stewart 2005	Analysis of evolution of status hierarchy in OSS community	x			
**128**	Teigland, Di Gangi 2014	Impact of boundary management of a firm-sponsored OSS community on community's innovation capacity and firm's absorptive capacity		x		
**129**	Terwiesch and Xu 2008	Decision on and design of innovation contest		x		
**130**	Toral, Martinez-Torres 2009	Characterization of mailing list in OSS development	x			
**131**	Toral, Martinez-Torres 2009	Factors influencing successful development of OSS virtual communities (from SNA perspective)	x			
**132**	Toubia and Laurent 2007	Development of a tool for involving conumers in idea screening		x		
**133**	van Dijk, Antonides 2014	Effect of consumer's awareness of co-creation on perceptions of brand personality and behavioural intentions	x			
**134**	Villarroel, Taylor 2013	Effects of different types and levels of knowledge sharing behavior on learning and innovation outcomes of competing communities	x		x	
**135**	Wei 2013	Categorization of web-based interactive innovation		x		

	Authors, Date	Research Focus	Category			
			User and community Factors	Organiza-tional Factors	Output and Outcome	Other
**136**	Weiber, Muhlhaus 2014	Interdependence between functional elements of OIC and the effects of motives and cognitive success dimensions on planned activities	**x**		**x**	
**137**	West and O'Mahony 2008	Creation and design of OIC and their effect on the ability to attract users	**x**	**x**		
**138**	Williams and Cothrel 2000	OIC as support for business startegy		**x**		
**139**	Xu, Jones 2009	Role of involvement on the developer's performance in an OSS project	**x**			
**140**	Xu, Lin 2011	Control mechanisms in OSS projects and their effect on project outcomes	**x**		**x**	
**141**	Yuxiang and Qinghua 2014	Motivations to participate in crowdsourcing contest	**x**			
**142**	Yücesan 2013	Operationalization of the screening process through a ranking and selection approach that is both efficient		**x**		
**143**	Zhang, Hahn 2013	Influence of community response and members' roles on continued partici-pation in OSS projects	**x**			
**144**	Zheng, Xie 2014	Impact of award and sponsor-solvers interaction on solution quantity, solution diversity, and solution quality		**x**	**x**	

References

1. Abdelkafi N, Blecker T, Raasch C. From open source in the digital to the physical world: a smooth transfer? Management Decision. 2009;47(10):1610-32.

2. Alavi S, Ahuja V, Medury Y. Metcalfe's law and operational, analytical and collaborative CRM-using online business communities for co-creation. Journal of Targeting, Measurement & Analysis for Marketing. 2012;20(1):35-45.

3. Antikainen M, Mäkipää M, Ahonen M. Motivating and supporting collaboration in open innovation. European Journal of Innovation Management. 2010;13(1):100-19.

4. Antorini YM, Muñiz JAM, Askildsen T. Collaborating With Customer Communities: Lessons from the Lego Group. MIT Sloan Management Review. 2012;53(3):73-95.

5. Antorini YM, Muñiz AM, Jr. The Benefits and Challenges of Collaborating with User Communities. Research Technology Management. 2013;56(3):21-8.

6. Balka K, Raasch C, Herstatt C. The Effect of Selective Openness on Value Creation in User Innovation Communities. Journal of Product Innovation Management. 2014;31(2):392-407.

7. Barcellini F, Detienne F, Burkhardt J-M. Participation in online interaction spaces: Design-use mediation in an Open Source Software community. International Journal of Industrial Ergonomics. 2009;39(3):533-40.

8. Battistella C, Nonino F. What drives collective innovation? Exploring the system of drivers for motivations in open innovation, Web-based platforms. Information Research. 2012;17(1); Paper 513. Available: http://InformationR.net/ir/17-1/paper513.html.

9. Battistella C, Nonino F. Open innovation web-based platforms: The impact of different forms of motivation on collaboration. Innovation : Management, Policy & Practice. 2012;14(4):557-75.

10. Battistella C, Nonino F. Exploring the impact of motivations on the attraction of innovation roles in open innovation web-based platforms. Production Planning & Control. 2013;24(2-3):226-45. doi: 10.1080/09537287.2011.647876

11. Bayus BL. Crowdsourcing New Product Ideas over Time: An Analysis of the Dell IdeaStorm Community. Management science. 2013;59(1):226-44.

12. Bianchi AJ, Kang SM, Stewart D. The Organizational Selection of Status Characteristics: Status Evaluations in an Open Source Community. Organization Science. 2012;23(2):341-54.

13. Birkinshaw J, Bouquet C, Barsoux J-L. The 5 Myths of Innovation. MIT Sloan Management Review. 2011;52(2):43-50.

14. Blohm I, Bretschneider U, Leimeister JM, Krcmar H. Does collaboration among participants lead to better ideas in IT-based idea competitions? An empirical investigation. International Journal of Networking & Virtual Organisations. 2011;9(2):106-22.

15. Bothos E, Apostolou D, Gregoris M. Collective intelligence for idea management with Internet-based information aggregation markets. Internet Research. 2009;19(1):26-41.

16. Boudreau KJ, Lacetera N, Lakhani KR. Incentives and Problem Uncertainty in Innovation Contests: An Empirical Analysis. Management science. 2011;57(5):843-63.

17. Boudreau KJ. Let a Thousand Flowers Bloom? An Early Look at Large Numbers of Software App Developers and Patterns of Innovation. Organization Science. 2012;23(5):1409-27.

18. Brabham DC. MOVING THE CROWD AT THREADLESS. Information, Communication & Society. 2010;13(8):1122-45.

19. Capra E, Francalanci C, Merlo F. An Empirical Study on the Relationship Between Software Design Quality, Development Effort and Governance in Open Source Projects. IEEE Transactions on Software Engineering. 2008;34(6):765-82.

20. Cheng J. How Macromedia Used Blogs to Build Its Developers' Communities: A Case Study. Performance Improvement Quarterly. 2008;21(3):43-58.

21. Cheng CCJ, Tsai H-T, Krumwiede D. How to enhance new product creativity in the online brand community? Innovation : Management, Policy & Practice. 2013;15(1):83-96.

22. Chua AYK, Banerjee S. Customer knowledge management via social media: the case of Starbucks. Journal of Knowledge Management. 2013;17(2):237-49.

23. Colombo MG, Piva E, Rossi-Lamastra C. Open innovation and within-industry diversification in small and medium enterprises: The case of open source software firms. Research Policy. 2014;43(5):891-902.

24. Cromie J, Ewing M. Squatting at the digital campfire - Researching the open source software community. International Journal of Market Research. 2008;50(5):631-53. doi: 10.2501/s1470785308200079

25. Dahlander L, Piezunka H. Open to suggestions: How organizations elicit suggestions through proactive and reactive attention. Research Policy. 2014;43(5):812-27.

26. Dahlander L. Penguin in a new suit: a tale of how de novo entrants emerged to harness free and open source software communities. Industrial and corporate change. 2007;16(5):913-43.

27. Dahlander L, Frederiksen L. The Core and Cosmopolitans: A Relational View of Innovation in User Communities. Organization Science. 2012;23(4):988-1007.

28. de Toni AF, Biotto G, Battistella C. Organizational design drivers to enable emergent creativity in web-based communities. Learning Organization. 2012;19(4):337-51.

29. di Gangi PM, Wasko M. Steal my idea! Organizational adoption of user innovations from a user innovation community: A case study of Dell Ideastorm. Decision Support Systems. 2009;48(1):303-12.

30. di Gangi PM, Wasko MM, Hooker RE. GETTING CUSTOMERS' IDEAS TO WORK FOR YOU: LEARNING FROM DELL HOW TO SUCCEED WITH ONLINE USER INNOVATION COMMUNITIES. Mis Quarterly Executive. 2010;9(4):213-28.

31. Djelassi S, Decoopman I. Customers' participation in product development through crowdsourcing: Issues and implications. Industrial Marketing Management. 2013;42(5):683-92.

32. Evans-Cowley J, Hollander J. The New Generation of Public Participation: Internet-based Participation Tools. Planning Practice & Research. 2010;25(3):397-408.

33. Fagerholm F, Sanchez Guinea A, Borenstein J, Munch J. Onboarding in Open Source Projects. IEEE Software. 2014;31(6):54-61.

34. Filieri R. Consumer co-creation and new product development: A case study in the food industry. Marketing Intelligence & Planning. 2013;31(1):40-53.

35. Fleming L, Waguespack DM. Brokerage, boundary spanning, and leadership in open innovation communities. Organization Science. 2007;18(2):165-80. doi: 10.1287/orsc.1060.0242

36. Franke N, Keinz P, Klausberger K. "Does This Sound Like a Fair Deal?": Antecedents and Consequences of Fairness Expectations in the Individual's Decision to Participate in Firm Innovation. Organization Science. 2013;24(5):1495-516.

37. Franke N, Hader C. Mass or Only 'Niche Customization'? Why We Should Interpret Configuration Toolkits as Learning Instruments. Journal of Product Innovation Management. 2014;31(6):1214-34.

38. Füller J, Jawecki G, Mühlbacher H. Innovation creation by online basketball communities. Journal of Business Research. 2007;60(1):60-71.

39. Füller J, Hutter K, Faullant R. Why co-creation experience matters? Creative experience and its impact on the quantity and quality of creative contributions. R & D Management. 2011;41(3):259-73.

40. Füller J, Hutter K, Hautz J, Matzler K. User Roles and Contributions in Innovation-Contest Communities. Journal of Management Information Systems. 2014;31(1):273-308.

41. Füller J, Matzler K, Hutter K, Hautz J. Consumers' Creative Talent: Which Characteristics Qualify Consumers for Open Innovation Projects? An Exploration of Asymmetrical Effects. Creativity & Innovation Management. 2012;21(3):247-62.

42. Füller J, Mühlbacher H, Matzler K, Jawecki G. Consumer Empowerment Through Internet-Based Co-creation. Journal of Management Information Systems. 2009;26(3):71-102.

43. Ghazawneh A. The power of platforms for software development in open innovation networks. International Journal of Networking & Virtual Organisations. 2011;9(2):140-54.

44. Grabher G, Ibert O. Distance as asset? Knowledge collaboration in hybrid virtual communities. Journal of Economic Geography. 2014;14(1):97-123.

45. Guimarães ALS, Korn HJ, Shin N, Eisner AB. The Life Cycle of Open Source Software Development Communities. Journal of Electronic Commerce Research. 2013;14(2):167-82.

46. Hann I-H, Roberts JA, Slaughter SA. All Are Not Equal: An Examination of the Economic Returns to Different Forms of Participation in Open Source Software Communities. Information Systems Research. 2013;24(3):520-38.

47. Hennala L, Parjanen S, Uotila T. Challenges of multi-actor involvement in the public sector front-end innovation processes. European Journal of Innovation Management. 2011;14(3):364-87.

48. Henttonen K, Pussinen P, Koivumäki T. Managerial Perspective on Open Source Collaboration and Networked Innovation. Journal of Technology Management & Innovation. 2012;7(3):135-47.

49. Hess J, Randall D, Pipek V, Wulf V. Involving users in the wild—Participatory product development in and with online communities. International Journal of Human-Computer Studies. 2013;71(5):570-89.

50. Hildebrand C, Häubl G, Herrmann A, Landwehr JR. When Social Media Can Be Bad for You: Community Feedback Stifles Consumer Creativity and Reduces Satisfaction with Self-Designed Products. Information Systems Research. 2013;24(1):14-29.

51. Huang Y, Singh PV, Srinivasan K. Crowdsourcing New Product Ideas Under Consumer Learning. Management science. 2014;60(9):2138-59.

52. Hutter K, Hautz J, Fueller J, Mueller J, Matzler K. Communitition: The Tension between Competition and Collaboration in Community-Based Design Contests. Creativity and Innovation Management. 2011;20(1):3-21.

53. Jarvenpaa SL, Tuunainen VK. How Finnair Socialized Customers for Service Co-Creation with Social Media. Mis Quarterly Executive. 2013;12(3):125-36.

54. Jeppesen LB, Frederiksen L. Why Do Users Contribute to Firm-Hosted User Communities? The Case of Computer-Controlled Music Instruments. Organization Science. 2006;17(1):45-63.

55. Jespersen KR. User-Involvement and open Innovation: The Case of Decision-maker Openness. International Journal of Innovation Management. 2010;14(3):471-89.

56. Jespersen KR. Online channels and innovation: Are users being empowered and involved? International Journal of Innovation Management. 2011;15(6): 1141-1159.

57. Jin B, Park JY, Kim H-S. What makes online community members commit? A social exchange perspective. 2010. p. 587-99.

58. Juell-Skielse G, Hjalmarsson A, Juell-Skielse E, Johannesson P, Rudmark D. Contests as innovation intermediaries in open data markets. Information Polity: The International Journal of Government & Democracy in the Information Age. 2014;19(3/4):247-62.

59. Khan Z, Ludlow D, Loibl W, Soomro K. ICT enabled participatory urban planning and policy development. Transforming Government: People, Process and Policy. 2014;8(2):205. doi: 10.1108/TG-09-2013-0030

60. Kim JH, Bae Z-T, Kang SH. The role of online brand community in new product development: Case studies on digital product manufacturers in Korea. International Journal of Innovation Management. 2008;12(3):357-76.

61. King A, Lakhani KR. Using Open Innovation to Identify the Best Ideas. MIT Sloan Management Review. 2013;55(1):41-8.

62. Koch S. Profiling an Open Source Project Ecology and Its Programmers. Electronic Markets. 2004;14(2):77-88.

63. Kohler T, Matzler K, Füller J. Avatar-based innovation: Using virtual worlds for real-world innovation. Technovation. 2009;29(6/7):395-407.

64. Kosonen M, Gan C, Olander H, Blomqvist K. My idea is our idea! Supporting user-driven innovation activities in crowdsourcing communities. International Journal of Innovation Management. 2013;17(3):1-18.

65. Kosonen M, Gan C, Vanhala M, Blomqvist K. User motivation and knowledge sharing in idea corwdsourcing. International Journal of Innovation Management. 2014;18(5). doi: 10.1142/s1363919614500315

66. Kuo-Ming C, Hui-Chun C. Community based innovation: its antecedents and its impact on innovation success. Internet Research. 2009;19(5):496-516.

67. Lampel J, Jha PP, Bhalla A. Test-Driving the Future: How Design Competitions Are Changing Innovation. Academy of Management Perspectives. 2012;26(2):71-85.

68. Latusek-Jurczak D, Prystupa-Rządca K. Collaboration and trust-building in open innovation community. Journal of Economics & Management. 2014(17):47-62.

69. Latzko-Toth G. Users as Co-Designers of Software-Based Media: The Co-Construction of Internet Relay Chat. Canadian Journal of Communication. 2014;39(4):577-95.

70. Lee H, Han J, Suh Y. Gift or threat? An examination of voice of the customer: The case of MyStarbucksIdea.com. Electronic Commerce Research and Applications. 2014;13(3):205-19.

71. Lee SM, Hwang T, Choi D. Open innovation in the public sector of leading countries. Management Decision. 2012;50(1):147-62.

72. Leimeister JM, Huber M, Bretschneider U, Krcmar H. Leveraging Crowdsourcing: Activation-Supporting Components for IT-Based Ideas Competition. Journal of Management Information Systems. 2009;26(1):197-224.

73. Lerner J, Pathak PA, Tirole J. The Dynamics of Open-Source Contributors. The American Economic Review. 2006;96(2):114-8.

74. Levine SS, Prietula MJ. Open Collaboration for Innovation: Principles and Performance. Organization Science. 2014;25(5):1414-33.

75. Liu H, Zhang J, Liu R, Li G. A model for consumer knowledge contribution behavior: the roles of host firm management practices, technology effectiveness, and social capital. Information Technology and Management. 2014;15(4):255-70.

76. Lüders M, Folstad A, Waldal E. Expectations and Experiences With MyLabourParty: From Right to Know to Right to Participate? Journal of Computer-Mediated Communication. 2014;19(3):446-62.

77. MacCormack A, Murray F, Wagner E. Spurring Innovation Through Competitions. MIT Sloan Management Review. 2013;55(1):25-32.

78. Malhotra A, Majchrzak A. Managing Crowds in Innovation Challenges. California Management Review. 2014;56(4):103-23.

79. Mallapragada G, Grewal R, Lilien G. User-Generated Open Source Products: Founder's Social Capital and Time to Product Release. Marketing Science. 2012;31(3): 474-492.

80. Marchi G, Giachetti C, de Gennaro P. Extending lead-user theory to online brand communities: The case of the community Ducati. Technovation. 2011;31(8):350-61.

81. Martinez-Torres MR. Analysis of activity in open-source communities using social network analysis techniques. Asian Journal of Technology Innovation. 2014;22(1):114-30.

82. Martinez-Torres MR. Application of evolutionary computation techniques for the identification of innovators in open innovation communities. Expert Systems with Applications. 2013;40(7):2503-10.

83. Martinez-Torres MR. Analysis of open innovation communities from the perspective of social network analysis. Technology Analysis & Strategic Management. 2014;26(4):435-51.

84. Martinez-Torres MR, Toral SL, Barrero F, Cortes F. The role of Internet in the development of future software projects. Internet Research. 2010;20(1):72-86.

85. Martini A, Massa S, Testa S. Customer co-creation projects and social media: The case of Barilla of Italy. Business horizons. 2014;57(3):425-34.

86. Martini A, Massa S, Testa S. The firm, the platform and the customer: A "double mangle" interpretation of social media for innovation. Information & Organization. 2013;23(3):198-213.

87. Martins CS, Patricio L. Understanding participation in company social networks. Journal of Service Management. 2013;24(5):567-87.

88. Mortara L, Ford SJ, Jaeger M. Idea Competitions under scrutiny: Acquisition, intelligence or public relations mechanism? Technological Forecasting and Social Change. 2013;80(8):1563-78.

89. Mount M, Martinez MG. Social Media: A Tool for Open Innovation. California Management Review. 2014;56(4):124-43.

90. Müller-Seitz G, Reger G. Networking beyond the software code? an explorative examination of the development of an open source car project. Technovation. 2010;30(11/12):627-34.

91. Nambisan S, Baron RA. Different Roles, Different Strokes: Organizing Virtual Customer Environments to Promote Two Types of Customer Contributions. Organization Science. 2010;21(2): 554-572.

92. Nambisan S, Nambisan P. How to Profit From a Better 'Virtual Customer Environment'. MIT Sloan Management Review. 2008;49(3):53-61.

93. Nätti S, Hurmelinna-Laukkanen P, Johnston WJ. Absorptive capacity and network orchestration in innovation communities - promoting service innovation. The Journal of Business & Industrial Marketing. 2014;29(2):173-84.

94. Ogawa S, Piller FT. Reducing the Risks of New Product Development. MIT Sloan Management Review. 2006;47(2):65-71.

95. Ogawa S, Pongtanalert K. Exploring Characteristics and Motives of Consumer Innovators. Research Technology Management. 2013;56(3):41-8.

96. Parjanen S, Hennala L, Konsti-Laakso S. Brokerage functions in a virtual idea generation platform: Possibilities for collective creativity? Innovation-Management Policy & Practice. 2012;14(3):363-74.

97. Paulini M, Murty P, Maher ML. Design processes in collective innovation communities: a study of communication. Codesign-International Journal of Cocreation in Design and the Arts. 2013;9(2):90-112.

98. Piller FT, Walcher D. Toolkits for idea competitions: a novel method to integrate users in new product development. R&d Management. 2006;36(3):307-18.

99. Poetz MK, Schreier M. The Value of Crowdsourcing: Can Users Really Compete with Professionals in Generating New Product Ideas? Journal of Product Innovation Management. 2012;29(2):245-56.

100. Raasch C. PRODUCT DEVELOPMENT IN OPEN DESIGN COMMUNITIES:: A PROCESS PERSPECTIVE. International Journal of Innovation & Technology Management. 2011;8(4):557-75.

101. Raasch C, Herstatt C, Balka K. On the open design of tangible goods. R & D Management. 2009;39(4):382-93.

102. Raasch C, von Hippel E. Innovation Process Benefits: The Journey as Reward. MIT Sloan Management Review. 2013;55(1):33-9.

103. Riedl C, Blohm I, Leimeister JM, Krcmar H. The Effect of Rating Scales on Decision Quality and User Attitudes in Online Innovation Communities. International Journal of Electronic Commerce. 2013;17(3):7-36. doi: 10.2753/jec1086-4415170301

104. Roberts DL, Candi M. Leveraging Social Network Sites in New Product Development: Opportunity or Hype? Journal of Product Innovation Management. 2014;31:105-17. doi: 10.1111/jpim.12195

105. Roberts D, Hughes M, Kertbo K. Exploring consumers' motivations to engage in innovation through co-creation activities. European Journal of Marketing. 2014;48(1/2):147-69.

106. Roberts N, Grover V. Leveraging Information Technology Infrastructure to Facilitate a Firm's Customer Agility and Competitive Activity: An Empirical Investigation. Journal of Management Information Systems. 2012;28(4):231-70.

107. Rossi C. Online consumer communities, collaborative learning and innovation. Measuring Business Excellence. 2011;15(3):46-62.

108. Russo-Spena T, Mele C. "Five Co-s" in innovating: a practice-based view. Journal of Service Management. 2012;23(4):527-53.

109. Ryzhkova N. Web-based customer innovation: A replication with extension. Innovation : Management, Policy & Practice. 2012;14(3):416-30.

110. Saldanha FP, Cohendet P, Pozzebon M. Challenging the Stage-Gate Model in Crowdsourcing: The Case of Fiat Mio in Brazil. Technology Innovation Management Review. 2014;4(9):28-35.

111. Sarkar S, Costa AIA. Dynamics of open innovation in the food industry. Trends in Food Science & Technology. 2008;19(11):574-80.

112. Sasinovskaya O, Anderson H. From brand awareness to online co-design: How a small bathroom provider turned interactive on the Web. Journal of Brand Management. 2011;19(1):33-44.

113. Saxton GD, Oh O, Kishore R. Rules of Crowdsourcing: Models, Issues, and Systems of Control. Information Systems Management. 2013;30(1):2-20.

114. Schlagwein D, Bjorn-Andersen N. Organizational Learning with Crowdsourcing: The Revelatory Case of LEGO. Journal of the Association for Information Systems. 2014;15(11):754-78.

115. Schweitzer FM, Buchinger W, Gassmann O, Obrist M. Crowdsourcing. Leveraging Innovation through Online Idea Competitions. Research Technology Management. 2012;55(3):32-8.

116. Scupola A, Hanne Westh N. Service innovation in academic libraries: is there a place for the customers? Library Management. 2010;31(4/5):304-18.

117. Scupola A, Nicolajsen HW. Using Social Media for Service Innovations: Challenges and Pitfalls. International Journal of E-Business Research. 2013;9(3):27-37.

118. Seltzer E, Mahmoudi D. Citizen Participation, Open Innovation, and Crowdsourcing: Challenges and Opportunities for Planning. Journal of Planning Literature. 2013;28(1):3-18.

119. Setia P, Rajagopalan B, Sambamurthy V, Calantone R. How Peripheral Developers Contribute to Open-Source Software Development. Information Systems Research. 2012;23(1): 144-163.

120. Shen C, Monge P. Who connects with whom? A social network analysis of an online open source software community. First Monday. 2011; 16(6). doi: 10.5210/fm.v16i6.3551

121. Sigala M. Social networks and customer involvement in new service development (NSD). International Journal of Contemporary Hospitality Management. 2012;24(7):966-90.

122. Singh PV. The Small-World Effect: The Influence of Macro-Level Properties of Developer Collaboration Networks on Open-Source Project Success. ACM Transactions on Software Engineering and Methodology. 2010;20(2). doi: 10.1145/1824760.1824763

123. Son J, Amrut S, Manchiraju S, Fiore AM, Niehm LS. Consumer adoption of online collaborative customer co-design. Journal of Research in Interactive Marketing. 2012;6(3):180-97.

124. Spaeth S, Stuermer M, Georg Von K. Enabling knowledge creation through outsiders: towards a push model of open innovation. International Journal of Technology Management. 2010;52(3,4):411-31.

125. Spiliotopoulou L, Charalabidis Y, N. Loukis E, Diamantopoulou V. A framework for advanced social media exploitation in government for crowdsourcing. Transforming Government: People, Process and Policy. 2014;8(4):545-568.

126. Stam W. When does community participation enhance the performance of open source software companies? Research Policy. 2009;38(8):1288-99.

127. Stewart D. Social status in an open-source community. American Sociological Review. 2005;70(5):823-42.

128. Teigland R, Di Gangi PM, Flaten BT, Giovacchini E, Pastorino N. Balancing on a tightrope: Managing the boundaries of a firm-sponsored OSS community and its impact on innovation and absorptive capacity. Information and Organization. 2014;24(1):25-47.

129. Terwiesch C, Jr., Xu Y. Innovation contests, open innovation, and multiagent problem solving. Management science. 2008;54(9):1529-43.

130. Toral SL, Martinez-Torres MR, Barrero F. Modelling Mailing List Behaviour in Open Source Projects: the Case of ARM Embedded Linux. Journal of Universal Computer Science. 2009;15(3):648-64.

131. Toral SL, Martinez-Torres MR, Barrero FJ. Virtual communities as a resource for the development of OSS projects: the case of Linux ports to embedded processors. Behaviour & Information Technology. 2009;28(5):405-19.

132. Toubia O, Laurent F. Adaptive Idea Screening Using Consumers. Marketing Science. 2007;26(3): 342-360.

133. van Dijk J, Antonides G, Schillewaert N. Effects of co-creation claim on consumer brand perceptions and behavioural intentions. International Journal of Consumer Studies. 2014;38(1):110-8.

134. Villarroel JA, Taylor JE, Tucci CL. Innovation and learning performance implications of free revealing and knowledge brokering in competing communities: insights from the Netflix Prize challenge. Computational and Mathematical Organization Theory. 2013;19(1):42-77.

135. Wei W. An empirically derived framework of web-based interactive innovation practices. Innovation-Management Policy & Practice. 2013;15(1):69-82.

136. Weiber R, Muhlhaus D, Kim JS, Hyun JH. Motives, success factors, and planned activities in a community of innovation as a critical mass system. Total Quality Management & Business Excellence. 2014;25(9-10):1105-25.

137. West J, O'Mahony S. The Role of Participation Architecture in Growing Sponsored Open Source Communities. Industry and innovation. 2008;15(2):145-68.

138. Williams RL, Cothrel J. Four Smart Ways to Run Online Communities. Sloan management review. 2000;41(4):81-91.

139. Xu B, Jones DR, Shao B. Volunteers' involvement in online community based software development. Information & Management. 2009;46(3):151-8.

140. Xu B, Lin Z, Xu Y. A Study of Open Source Software Development from Control Perspective. Journal of Database Management. 2011;22(1):26.

141. Yuxiang CZ, Qinghua Z. Effects of extrinsic and intrinsic motivation on participation in crowdsourcing contest. Online Information Review. 2014;38(7): 896-917.

142. Yücesan E. An efficient ranking and selection approach to boost the effectiveness of innovation contests. Iie Transactions. 2013;45(7):751-62. doi: 10.1080/0740817x.2012.757679

143. Zhang C, Hahn J, De P. Continued Participation in Online Innovation Communities: Does Community Response Matter Equally for Everyone? Information Systems Research. 2013;24(4): 1112-1130.

144. Zheng H, Xie Z, Hou W, Li D. Antecedents of solution quality in crowdsourcing: The sponsor's perspective. Journal of Electronic Commerce Research. 2014;15(3):212-24.
